# Caregivers’ Perceptions of Clinical Symptoms, Disease Management, and Quality of Life Impact in Cases of Cyclin-Dependent Kinase-Like 5 Deficiency Disorder: Cross-Sectional Online Survey

**DOI:** 10.2196/72489

**Published:** 2025-06-10

**Authors:** Sam Amin, Carol-Anne Partridge, Helen Leonard, Jenny Downs, Helen Allvin, Valentine Ficara, Emilie Pain, Minna A Korolainen

**Affiliations:** 1 Department of Paediatric Neurology Bristol Royal Hospital for Children Bristol United Kingdom; 2 CDKL5 UK Bristol United Kingdom; 3 The Kids Research Institute Australia The Centre for Child Health Research University of Western Australia Perth Australia; 4 Orion Corporation Orion Pharma Espoo Finland; 5 Carenity Paris France

**Keywords:** CDKL5 deficiency disorder, cyclin-dependent kinase-like 5, developmental epileptic encephalopathy, caregiver perception, burden, cross-sectional survey, online survey, health-related quality of life, epilepsy, rare disorder

## Abstract

**Background:**

Cyclin-dependent kinase-like 5 (CDKL5) deficiency disorder (CDD) is an ultrarare genetic condition causing developmental epileptic encephalopathy characterized by seizures and motor and intellectual disabilities. No disease-modifying therapies are available, and treatments focus mainly on symptom management to improve quality of life.

**Objective:**

The aim of this study was to better understand the burden of CDD based on family caregivers’ perceptions.

**Methods:**

The study was a cross-sectional, web-based survey comprising 40 questions for caregivers of patients with CDD and focusing on sociodemographic and medical characteristics, disease burden, unmet needs, treatments, and support. An adapted version of the EQ-5D-5L instrument was included to measure patients’ health-related quality of life as perceived by their caregivers.

**Results:**

A total of 132 caregivers, mostly from western parts of Europe, responded. The median patient age was 7.6 (IQR 2.9-12.2) years. Seizure onset occurred early, with the median onset at 2.0 (IQR 1.0-3.0) months of age. The median age at diagnosis was 1.2 (IQR 0.6-4.0) years. Epilepsy (123/132, 93.2%) and limited communication skills (111/132, 84.1%) were the most commonly reported symptoms. The highest number of different types of symptoms was reported for patients aged 5-9 years, with a median of 9.0 (IQR 7.5-10.0) symptoms. Most patients with epilepsy experienced daily seizures (81/123, 65.9%), and nearly all (119/123, 96.7%) were on antiseizure medications. A minority was on a ketogenic diet (21/123, 17.1%) or underwent vagus nerve stimulation (14/123, 11.4%). The care received was multidisciplinary. Compared to younger patients, adults had fewer medical appointments and a smaller variety of health care professionals in their care team. The EQ-5D-5L, adapted for caregivers, indicated low health-related quality of life for patients, with a median global index value of 0.18 (IQR 0.11-0.32). The most severe consequences of CDD on patients’ daily lives were reported for mobility (88/132, 66.7%), self-care (120/132, 90.9%), and everyday activities (103/132, 78.0%). Caregiver burden was also substantial, with all life aspects reportedly impacted by CDD, including professional life and financial resources (median impact ratings of 9.0/10 and 7.0/10, respectively). Access to support and care varied depending on location. Caregivers outside Europe reported a longer time between the first seizure and diagnosis (26.5, IQR 3.2-47.0 months) compared to European caregivers (11.0, IQR 5.0-45.0 months). They also reported a higher impact of CDD on their financial resources (rating of 10/10) compared to European caregivers (rating of 6/10) and greater challenges in covering costs.

**Conclusions:**

The study findings provide valuable insights on symptoms and disease burden related to CDD. This burden was quantitatively characterized with the EQ-5D-5L for the first time and was perceived as substantial by family caregivers. Discrepancies between geographic regions and age groups were highlighted, especially regarding available support and access to resources and care.

## Introduction

### Background

Cyclin-dependent kinase-like 5 (CDKL5) deficiency disorder (CDD) is an ultrarare genetic condition, with an estimated incidence of 2.36 per 100,000 live births [1]. It is a developmental epileptic encephalopathy condition and was first described in 2004 [2]. Initially, CDD was believed to be an early-onset variant of Rett syndrome, another rare neurological disorder, due to the similarity in symptoms (seizures, developmental delay, gastrointestinal dysfunction, scoliosis, etc). However, CDD has a distinct genetic cause and symptom profile, as severe developmental delay is exhibited from birth and seizure onset occurs earlier, and it was thus established as a separate clinical condition in 2013 [3-5]. CDD is caused by pathogenic variants in the X-linked gene *CDKL5*, which plays a key role in normal brain development and function [6]. The disorder is estimated to be 4 times more prevalent in females than in males, but male patients often experience more severe symptoms than their female counterparts [7].

CDD is characterized by a broad range of clinical features, including early-onset refractory epilepsy; hypotonia; developmental, motor, and intellectual disabilities; cerebral (cortical) visual impairment; and microcephaly [3]. Epileptic seizures are usually the first symptom to occur, with a median age of onset of 6 weeks (by the age of 3 months in 90% of cases) [3,8,9]. Psychomotor impairments, hypotonia, and significant intellectual disability in children with CDD result in difficulties with gross motor skills, such as sitting, standing, or walking, and communication skills, which are often limited to body language, facial expressions, and vocalizations [5,10-12]. Other common comorbidities of CDD include sleep disturbances, movement disorders, respiratory issues, and gastrointestinal disorders [13,14].

CDD can be suspected through clinical examination, but genetic testing is required to confirm the diagnosis [3]. Phenotypic variability exists between individuals in terms of symptom range and severity [3,7,15]. Management of CDD typically starts with pharmacological interventions to alleviate seizures and other comorbid symptoms, as no disease-modifying therapies are currently available [3,16,17]. Antiseizure medications (ASMs) are prescribed based on seizure types and patterns, with polypharmacy often necessary due to seizure refractoriness [3,18]. The most commonly used ASMs for CDD include levetiracetam, topiramate, sodium valproate, vigabatrin, phenobarbital, and clobazam [18]. Ganaxolone, a neuroactive steroid, demonstrated long-term reduction of CDD-associated seizure frequency in clinical trials and became the first ASM approved by US and European regulatory agencies for the treatment of seizures associated with CDD in patients aged 2 years or older [19-22]. In addition to medications, a ketogenic diet and vagus nerve stimulation (VNS; for patients over 4 years old) may also help to reduce the frequency and intensity of seizures [17,23,24]. For developmental and social issues, a multidisciplinary approach is considered essential, including speech therapy, physiotherapy, psychosocial support, and occupational therapy, to address the broader needs of patients [17]. Given the symptom load and the necessary care, the burden of CDD is considered substantial for patients and their family members. Families caring for children with CDD experience significant emotional and financial burdens, with primary caregivers, particularly mothers, reporting lower mental well-being. The severity of the child’s sleep disturbances and the financial hardships further exacerbate stress levels, while the significant care needs likely contribute to a higher caregiving burden and reduced family quality of life [25]. Emotional well-being appears to be more impacted in the parents of children with CDD than in those caring for children with other genetic disorders associated with intellectual disability, such as Down syndrome or Rett syndrome [26]. Although recent studies have provided major insights on the clinical features and disease management of CDD, research quantifying and qualifying this burden remains limited, given the scarcity of the disorder and its recent identification [14,25-29].

### Goal

The aim was to better understand the burden of CDD on both patients and their family caregivers and identify unmet needs by analyzing the perspectives of family caregivers in an international cross-sectional survey.

## Methods

### Study Design

This study included a descriptive, cross-sectional, web-based, self-administered survey completed by the caregivers of patients with CDD. The survey was designed and approved by a multidisciplinary steering committee of CDD specialists and caregivers.

### Study Population

Participants were recruited through CDD patient advocacy organizations, including CDKL5 UK (United Kingdom), CDKL5 Alliance Francophone (France), CDKL5 Deutschland (Germany), Asociacion de Afectados CDKL5 (Spain), CDKL5 Ireland, CDKL5 Italy, CDKL5 Alliance (World), Finnish Epilepsy Association (Finland), Svenska Epilepsiförbundet - Swedish association from the International Bureau for Epilepsy (IBE), and EpiCare-network (Europe), as well as through Carenity, a web-based community in which both patients and caregivers can share their experiences, provide support, and receive information. CDD caregivers were recruited via digital communication and invited by patient organizations via email to voluntarily complete an online survey about the burden of this disease and its impact on the quality of life of patients with CDD and their caregivers. Eligible participants included adult legal representatives or guardians (18 years or older at the time of inclusion) of individuals with genetically confirmed CDD. While the study initially targeted caregivers living in western parts of Europe, no respondents were excluded based on their country of residence. All participants provided electronic informed consent prior to participating in the survey.

### Survey and Data Collection

The questionnaire consisted of 3 sections with a total of 40 questions: sociodemographic and medical characteristics (25 questions); quality of life, disease burden, and unmet needs (10 questions); and treatment and support received (5 questions) (Multimedia Appendix 1). There were various question formats, such as single- and multiple-choice questions, numerical questions, slider-scale questions, and open-ended questions, and there was an adapted version of the self-administered EQ-5D-5L instrument, developed by the EuroQol Research Foundation to measure patients’ health-related quality of life [30,31]. The estimated completion time for the survey was 25 minutes, and the average completion time was 24.5 minutes.

The EQ-5D-5L proxy version 1 questionnaire was modified for caregivers to describe patients’ health state. The adapted version was validated by EuroQol [31]. Using a 5-level scale (no problems, slight problems, moderate problems, severe problems, and extreme problems), caregivers reported the severity of issues patients faced across 5 dimensions: mobility, self-care, everyday activities, pain/discomfort, and anxiety/depression. These levels are combined to obtain a single index value, reflecting the preferences of the general population in the caregiver’s country, with each dimension weighted accordingly (Multimedia Appendix 2) [32,33]. Additionally, using a visual analog scale (VAS), caregivers rated the patient’s health on the day the questionnaire was completed, ranging from 0 (worst health imaginable) to 100 (best health imaginable).

The impacts of CDD on the caregiver’s family, social, and work life; financial resources; quality of sleep; level of stress; and general quality of life were self-rated using a slider scale, with responses ranging from “no impact at all” (value=0) to “very high impact” (value=10).

The data collection occurred from May 25, 2023, to December 31, 2023.

### Statistical Analysis

Data management and analysis were performed using R (version 4.1.2; R Core Team).

A comprehensive quality check was performed prior to analysis on fully completed questionnaires in order to exclude respondents with inconsistent answers (eg, conflicting responses between similar questions, unrealistic values in numerical fields, or incoherent open-ended responses), response times shorter than the calculated minimum for the shortest completion path (2.6 minutes or less than 5 seconds per question), and suspected duplicate entries.

We conducted descriptive analyses to characterize our study population and summarize responses to the survey. The data have been presented as median and IQR or frequency and percentage values as appropriate. Responses to open-ended questions were translated into English when necessary and manually analyzed to identify categories and subcategories. Where relevant, subgroup analyses were conducted based on caregivers’ sociodemographic characteristics and patients’ medical characteristics.

### Ethical Considerations

This study was conducted in accordance with the current version of the Declaration of Helsinki, the ICH Good Epidemiological Practice guidelines, any local regulations, and the General Data Protection Regulation (GDPR). The study did not undergo an ethical review as it was considered as “a satisfaction survey” with a focus to better understand the impact of a disease (CDD) on patients and caregivers [34].

All patients provided electronic informed consent (translated to the language of the questionnaire) before any patient data collection. Participation was voluntary, and participants received no incentives to participate in the study.

## Results

### Participant Characteristics

**Figure 1:**
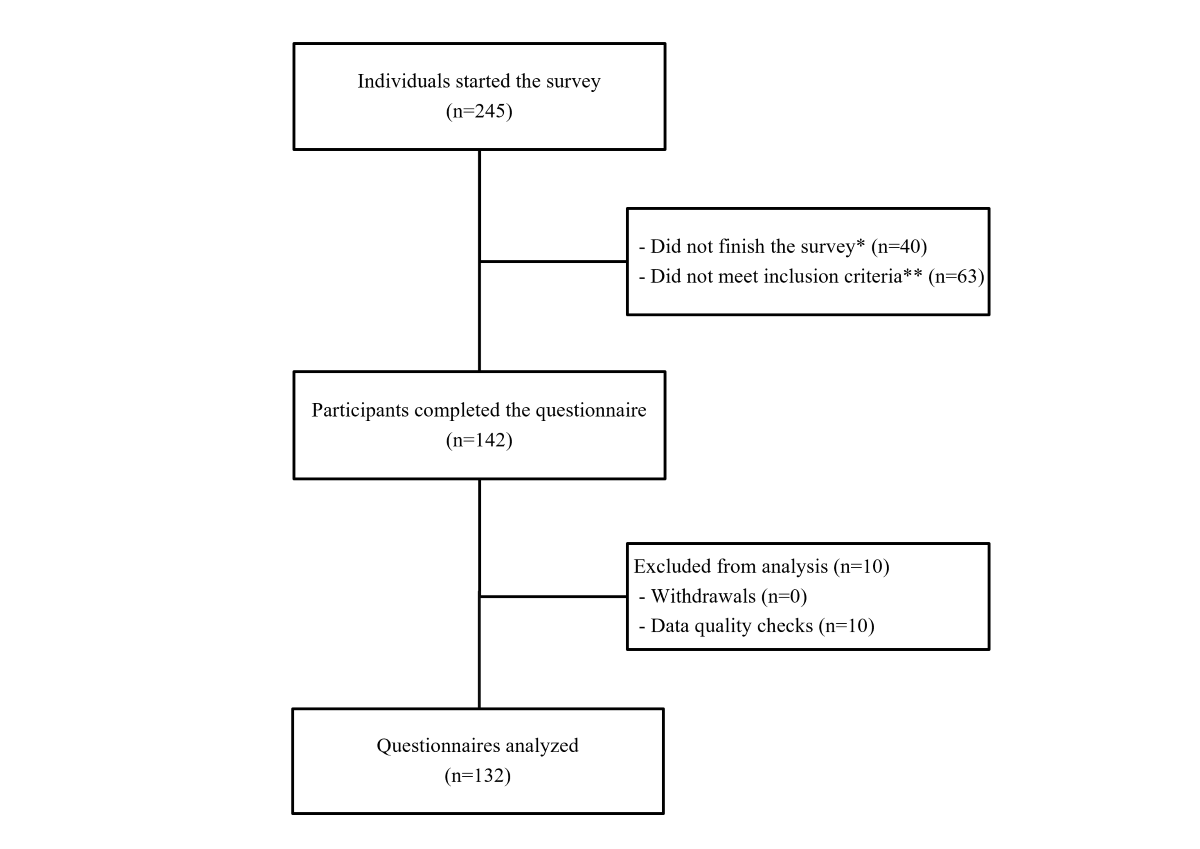
Flow diagram outlining the survey population of caregivers of patients with cyclin-dependent kinase-like 5 deficiency disorder (CDD): number of individuals who started the survey, number of individuals who were excluded during the questionnaire completion or before analysis, and final number of questionnaires analyzed. *Reasons for not finishing the survey cannot be precisely determined, and they may include individuals who started the survey out of curiosity; **Not a caregiver of a patient with CDD (n=38), automatically screened out after entering the child’s date of birth instead of the caregiver’s date of birth (n=11), not the legal guardian of the patient they cared for (n=10), and no genetic testing performed (n=4).

Of 245 participants who started the survey, 132 (53.9% response rate) completed a valid questionnaire (Figure 1). Most participants were from Europe (106/132, 80.3%), more specifically western Europe (France and Benelux; 32/132, 24.2%), southern Europe (Spain, Italy, and Portugal; 30/132, 22.7%), central Europe (Germany, Austria, and Switzerland; 21/132, 15.9%), and northern Europe (United Kingdom, Ireland, and Sweden; 20/132, 15.2%). A few responses were gathered from eastern Europe (Poland, Slovakia, and Montenegro; 4/132, 3.0%). Respondents outside Europe (26/132, 19.7%) were from Latin America (Argentina, Bolivia, Chile, Colombia, Peru, Mexico, and Uruguay; 15/132, 11.4%), Turkey (8/132, 6.1%), Australia (2/132, 1.5%), and the United States (1/132, 0.8%).

#### Sociodemographic Characteristics of Family Caregivers

Caregivers’ demographics are summarized in Table 1. All 132 respondents were parents of patients with CDD, with a median age of 41 years (IQR 35.0-46.0 years), and most were women (112/132, 84.8%). Most caregivers (85/132, 64.4%) did not work or worked part-time because of their children’s CDD. Caregivers outside Europe were slightly more likely to have their employment status impacted by CDD (19/26, 73%; either working part-time or not working because of CDD) compared to European caregivers (66/106, 62.3%).

**Table 1:** Sociodemographic characteristics of caregivers caring for patients with cyclin-dependent kinase-like 5 deficiency disorder.

Characteristic			Value (N=132)
**Gender, n (%)**			
	Female	112 (84.8)	
	Male	19 (14.4)	
	Did not answer	1 (0.8)	
Age (years)^a^, median (IQR)			41.0 (35.0-46.0)
**Age categories^a^, n (%)**			
	<40 years	57 (43.8)	
	≥40 years	73 (56.2)	
**Relationship with the patient, n (%)**			
	Parent	132 (100)	
**Caregiver’s role, n (%)**			
	Main caregiver	131 (99.2)	
	Not the main caregiver	1 (0.8)	
**Employment status, n (%)**			
	Working full time	32 (24.2)	
	Working part-time for reasons unrelated to CDD^b^	9 (6.8)	
	Working part-time because of CDD	39 (29.5)	
	Not working for reasons unrelated to CDD	4 (3.0)	
	Not working because of CDD	46 (34.8)	
	Other^c^	2 (1.5)	

^a^N=130; 2 answers were removed after data quality check.

^b^CDD: cyclin-dependent kinase-like 5 deficiency disorder.

^c^Other (n=2): working from home (n=1) and 99% reduction in working hours (n=1).

#### Sociodemographic Characteristics of Patients

Most patients with CDD were female (117/132, 88.6%), and two-thirds (88/131, 67.2%) were under 10 years old, with age ranging from 4 months to 38 years (Table 2). Patients in and outside Europe had similar demographic characteristics, although European patients were slightly older, with a median age of 7.7 years (IQR 3.0-14.4 years) compared to a median age of 5.2 years (IQR 1.9-8.2 years) for those living outside Europe. Most patients (127/132, 96.2%) lived in their family home.

**Table 2:** Sociodemographic characteristics of patients with cyclin-dependent kinase-like 5 deficiency disorder.

Characteristic		Value (N=132)
**Gender, n (%)**		
	Female	117 (88.6)
	Male	15 (11.4)
Age (years)^a^, median (IQR)		7.6 (2.9-12.2)
**Age categories^a^, n (%)**		
	<5 years	49 (37.4)
	≥5 and <10 years	39 (29.8)
	≥10 and <18 years	23 (17.6)
	≥18 years	20 (15.3)
**Living situation, n (%)**		
	Family home	127 (96.2)
	Specialized institution	3 (2.3)
	Community home	2 (1.5)
**Location, n (%)**		
	Central Europe (Germany, Austria, and Switzerland)	21 (15.9)
	Eastern Europe (Poland, Slovakia, and Montenegro)	4 (3.0)
	Northern Europe (United Kingdom, Ireland, and Sweden)	20 (15.6)
	Southern Europe (Spain, Italy, and Portugal)	30 (22.7)
	Western Europe (France and Benelux)	32 (24.2)
	Latin America (Argentina, Bolivia, Chile, Colombia, Peru, Mexico, and Uruguay)	15 (11.4)
	Turkey	8 (6.1)
	Other (Australia and United States)	3 (2.3)

^a^N=131; 1 answer was removed after data quality check.

### CDD Journey for Patients and Caregivers

#### Patients’ Medical Characteristics and Diagnoses

At inclusion, the median disease duration (time since the appearance of the first symptoms) was 7 years (IQR 2.7-12.3 years) and was longer for European patients (7.5 years, IQR 2.8-13.9 years) than for patients outside Europe (4.8 years, IQR 1.7-7.7 years). Nearly half (59/132, 44.7%) of the patients had access to a medical center specialized in CDD care, while the other half (61/132, 46.2%) did not. Half of the patients in Europe (54/106, 50.9%) had access to specialized centers compared to only 19% (5/26) of those outside Europe. The median time since diagnosis was 4.4 years (IQR 1.8-8.2 years).

All included patients had a genetically confirmed diagnosis, and the median age at diagnosis was 1.2 years (IQR 0.6-4.0 years), while the median age at the first seizure or symptoms was 2.0 months (IQR 1.0-3.0 months). Most patients (127/132, 96.2%) experienced their first CDD symptoms (seizures) before 12 months of age. No difference was observed based on geographic location, except for the median age at diagnosis, which was higher for patients outside Europe (2.7 years, IQR 0.5-4.0 years) than for European patients (1.1 years, IQR 0.6-4.0 years).

Medical attention for CDD symptoms was sought for a majority of patients (123/132, 93.2%) after the first seizure, with little to no delay (median 0.0 months). However, the median time between the first seizure or symptoms and diagnosis was 11 months (IQR 4.0-45.5 months). This time was longer for patients outside Europe (26.5 months, IQR 3.2-47.0 months) than for European patients (11 months, IQR 5.0-45.0 months) (Figure S1 in Multimedia Appendix 3).

**Figure 2:**
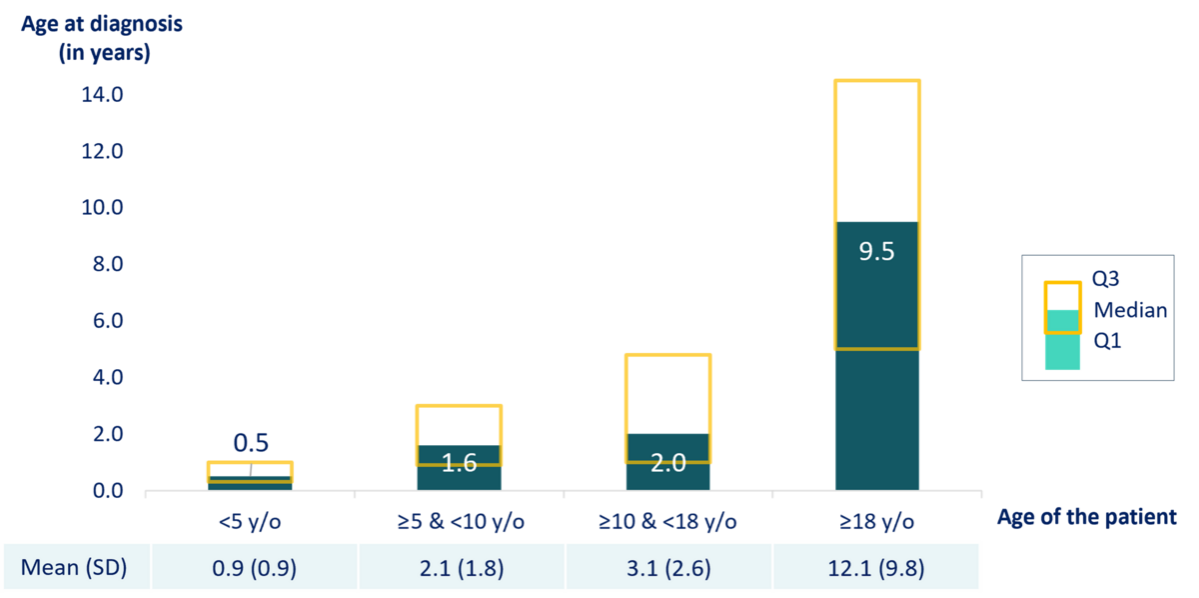
Mean and median age at diagnosis (in years) by age group at inclusion among patients with cyclin-dependent kinase-like 5 deficiency disorder (N=132). Patients are categorized into age subgroups: <5 years (n=48), ≥5 and <10 years (n=39), ≥10 and <18 years (n=23), and ≥18 years (n=20). Q1: first quartile; Q3: third quartile; y/o: years old.

The median age at diagnosis increased with the patient age at the time of the study (Figure 2).

#### CDD Symptoms

**Figure 3:**
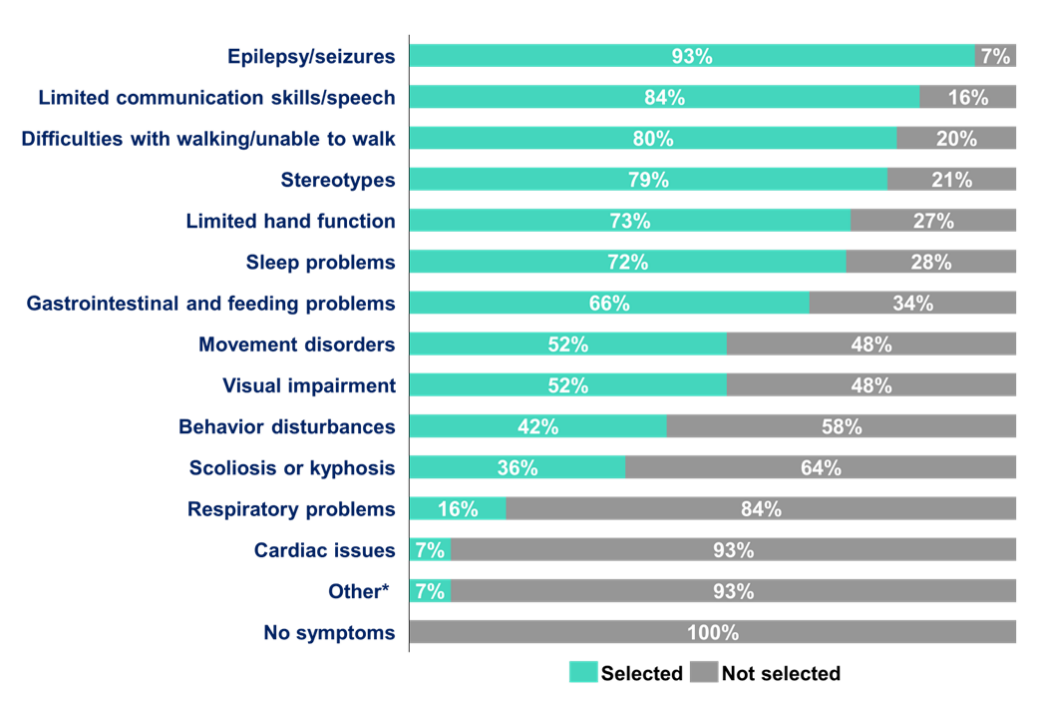
Cyclin-dependent kinase-like 5 deficiency disorder symptoms experienced over the past year as reported by caregivers. Percentage of patients who experienced the symptoms in the last 12 months (N=132). *Other (n=9): developmental disorder (n=1), scratching (n=1), hyperactivity (n=1), immune system issues (n=1), precocious puberty (n=1), kidney stones (n=1), hypotonia (n=1), osteoporosis (n=1), and very intense outbreaks of aggression (n=1).

All patients experienced at least one symptom over the past year, and 56.8% (75/132) experienced 8 or more symptoms (Figure 3). Epilepsy or seizures were reported in 93.2% (123/132) of patients, functional disabilities (eg, limited communication skills, difficulty with walking, stereotypies, limited hand function, movement disorders, visual impairment, and behavior disturbances) in 97.7% (129/132), and other medical problems (sleep problems, gastrointestinal and feeding problems, scoliosis or kyphosis, respiratory problems, and cardiac issues) in 89.4% (118/132). Patients aged 5-9 years experienced more symptoms, with a median of 9.0 (IQR 7.5-10.0) compared to older and younger age groups. Patients aged 10-17 years had a median of 8.0 symptoms (IQR 6.5-10.0), similar to adult patients (IQR 5.8-9.0). Patients aged <5 years had a median of 7.0 symptoms (IQR 7.0-9.0). Notably, 74% (29/39) of patients aged 5-9 years experienced 8 or more symptoms compared to only 39% (19/48) of patients aged <5 years.

Of 123 respondents who reported epilepsy or seizures, 120 (97.6%) were able to identify at least one type of seizure over the past year. The majority (99/120, 82.5%) reported multiple types, with a median of 3.0 different types (IQR 2.0-4.0). Patients aged 5-9 years had the most varied seizure types, with 23% (8/35) experiencing 5 or more types compared to 10% (2/21) of those aged 10-17 years, 11% (5/45) of those aged <5 years, and 11% (2/18) of those aged ≥18 years.

The most commonly reported type of seizure over the past year was major motor seizure (91/123, 74.0%), followed by epileptic spasms (81/123, 65.9%), absence seizure (71/123, 57.7%), and myoclonic seizure (59/123, 48.0%). Hypermotor-tonic-spasm sequence was the least reported type of seizure (43/123, 35.0%).

Two-thirds (81/123, 65.9%) of patients with epilepsy or seizures experienced daily seizures over the past month, and 27 (22.0%) had >5 seizures per day. For only 8 patients (6.5%), no seizures were reported in the month prior to completing the survey.

Patients aged 5-18 years were identified as having more frequent seizures compared to younger and older patients. We found that 83% (29/35) of patients aged 5-9 years and 77% (16/21) of those aged 10-17 years experienced daily seizures, whereas only 42% (8/19) of patients aged ≥18 years and 60% (28/47) of patients aged <5 years experienced daily seizures.

#### Treatments

Almost all patients with seizures (119/123, 96.7%) were receiving ASMs on the day caregivers completed the questionnaire, with a median of 2 medications per day (IQR 2.0-3.0). Patients aged 5-9 years were taking more daily ASMs, with a median of 3.0 (IQR 2.0-4.0) compared to other age groups (2.0, IQR 2.0-3.0). 

The number of daily ASMs taken tended to correlate positively with the frequency of seizures (Figure S2 in Multimedia Appendix 3). Patients experiencing >5 seizures per day took a median of 2.5 ASMs daily, and those with 1-5 seizures per day had a median of 3.0 ASMs. In contrast, patients who had experienced weekly, monthly, or no seizures over the past month had a lower median of 2.0 daily medications. Notably, 73% (24/33) of patients aged 5-9 years were on three or more daily ASMs compared to approximately 40% of patients in all other age groups.

Most respondents (108/132, 81.8%) reported discontinuing at least one ASM since diagnosis, with a median of 4.0 (IQR 2.0-7.0) ASMs stopped. The number was higher for older patients and for those with higher seizure frequency. A median of 5 ASMs had been discontinued for patients experiencing daily seizures (either 1-5 or >5), while those with weekly or monthly seizures discontinued only 2 ASMs.

At the time of the survey, only 17.1% (21/123) of patients were on a ketogenic diet and 11.4% (14/123) were on VNS. The percentage of patients on a ketogenic diet was consistent across regions, with 17% (16/97) in Europe and 19% (5/26) outside Europe. However, VNS was reported for 14% (14/97) of European patients, while none (0/26) of the patients outside Europe had undergone this treatment (Figure S3 in Multimedia Appendix 3). Additionally, differences were seen within Europe as well regarding VNS treatment, as it was not reported for any patient living in southern Europe (0/27; Italy, Spain, and Portugal). Patient age had a clear impact on the types of treatments received. Among patients aged <5 years, 34% (16/47) were on a ketogenic diet compared to only 6% (2/35) among those aged 5-9 years, 14% (3/21) among those aged 10-17 years, and none among those aged ≥18 years. VNS is not recommended for children aged <4 years, and it was not reported for any patient aged <5 years [23]. However, VNS was used in 11% (4/35) of patients aged 5-9 years, 29% (6/21) of those aged 10-17 years, and 21% (4/19) of those aged ≥18 years.

Seizure frequency did not influence the number of patients on a ketogenic diet, but the use of VNS tended to correlate positively with seizure frequency as follows: 8% (1/12) of patients with a few seizures per month, 9% (2/22) of those with weekly seizures, 11% (6/54) of those with 1-5 seizures a day, and 19% (5/27) of those with >5 seizures a day.

More than half (75/123, 61.0%) of the caregivers of patients with seizures reported not using any rescue medications during the past year. This percentage was higher among the caregivers of younger patients (33/47, 70% for those aged <5 years) compared to other age groups (Figure S4 in Multimedia Appendix 3).

**Figure 4:**
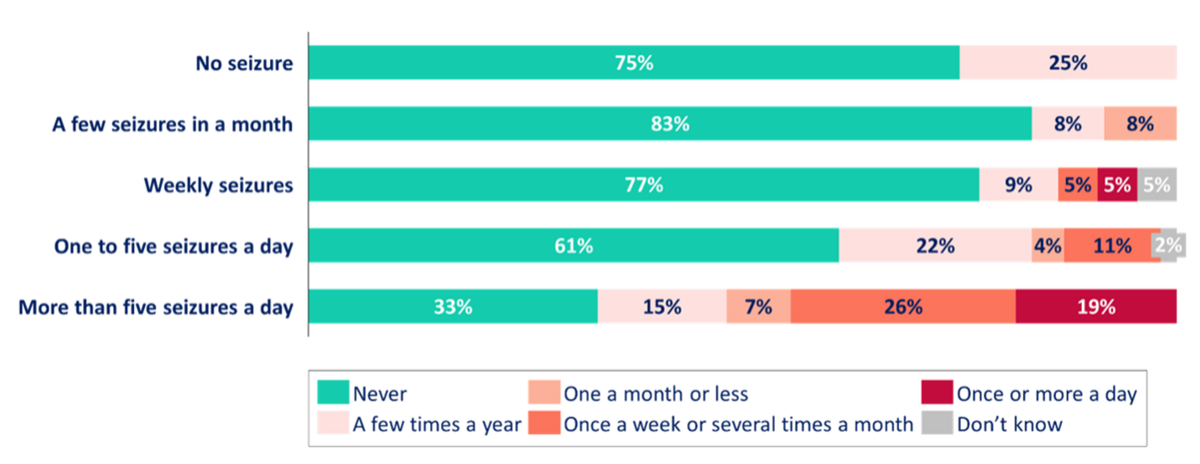
Frequency of rescue medication use in the past year by frequency of seizures experienced over the past month by patients with cyclin-dependent kinase-like 5 deficiency disorder. Patients who experienced seizures (n=123) were categorized into subgroups according to seizure frequency over the past year: no seizure (n=8), a few seizures a month (n=12), weekly seizures (n=22), 1-5 seizures a day (n=54), and >5 seizures a day (n=27). The frequency of rescue medication is represented in percentage within each category of seizure frequency.

The use of rescue medications tended to be higher in patients with higher seizure frequency (Figure 4). Among the caregivers of patients with >5 seizures a day, 67% (18/27) used rescue medications at least once in the past year compared to 37% (20/54) of those with 1-5 seizures a day, 18% (4/22) of those with weekly seizures, and 17% (2/12) of those experiencing only a few seizures per month. Rescue medications were also used more frequently in groups with higher seizure frequency, with 44% (12/27) of caregivers of patients with >5 seizures a day using them several times a month or more often. This number did not exceed 11% in any other group.

Patients who had discontinued fewer ASMs received rescue medications less frequently in the past year. Only 25% (3/12) of those who had not stopped any medications and 29% (13/45) of those who had stopped up to four medications reported using rescue medications. In contrast, the frequency of rescue medication use was higher among patients who had discontinued more medications. We found that 49% (19/39) of those who had stopped 5-10 medications and 44% (8/18) of those who had stopped ≥10 medications had used rescue medications in the past year.

Family caregivers of patients treated for epileptic seizures (n=119) reported dissatisfaction with both the effectiveness of ASMs in reducing seizures, rating it a median of 4 out of 10 (IQR 1.8-7.0), and the side effects (4/10, IQR 2.0-7.0). However, satisfaction regarding the burden of administration was slightly higher, with a median rating of 5.5 out of 10 (IQR 3.0-8.2).

**Figure 5:**
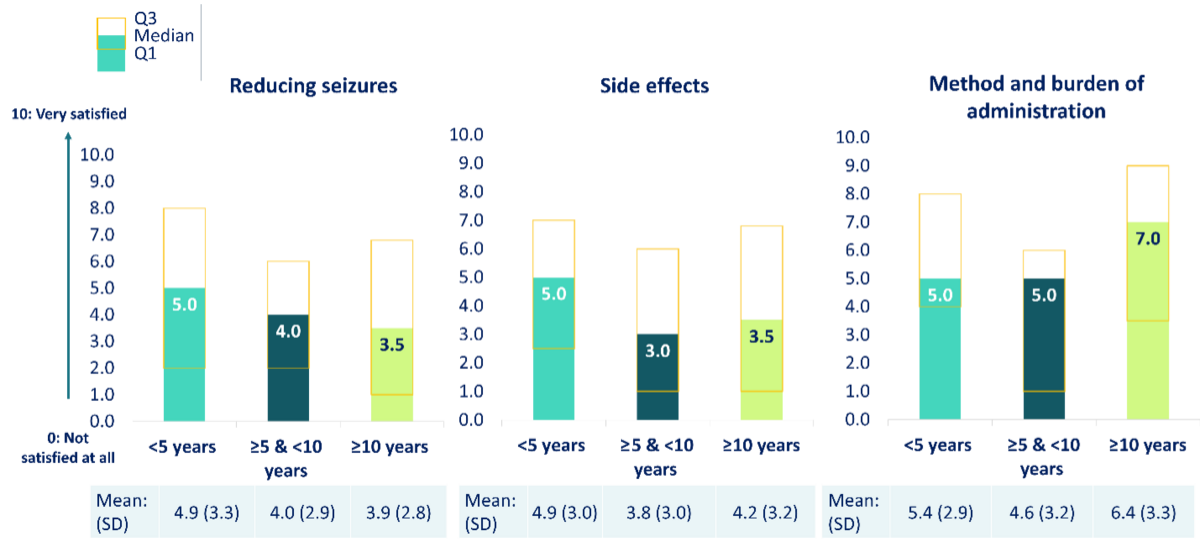
Mean and median caregiver satisfaction with the effectiveness, side effects, treatment method, and burden of administration of seizure-reducing treatments by time since the first seizure or symptoms, reported on a scale from 0 (not satisfied at all) to 10 (very satisfied) (n=118). Patients with cyclin-dependent kinase-like 5 deficiency disorder were categorized into subgroups according to the time since their first seizure or symptoms: <5 years (n=47), ≥5 and <10 years (n=33), and ≥10 years (n=38). Q1: first quartile; Q3: third quartile.

Satisfaction with the effectiveness of seizure-reducing treatments and side effects decreased with time since the onset of the first seizure or symptoms (or time since disease onset) in contrast to satisfaction with the method and administration of treatment, which increased with time (Figure 5).

Satisfaction with treatment effectiveness decreased with the frequency of seizures experienced over the past month. Caregivers gave a median rating of 9 out of 10 (IQR 8.0-10.0) for patients with no seizures, 5 out of 10 for patients with a few seizures per month (IQR 2.5-8.0) and those with weekly seizures (IQR 4.0-7.0), and 3 out of 10 (IQR 0.2-4.8) for patients with daily seizures.

#### Health Care Appointments

Most patients (81/132, 61.4%) had appointments with 6 or more different types of health care specialists over the past year, with a median of 7.0 (IQR 5.0-8.0) different health care professionals involved (Figure S5 in Multimedia Appendix 3). The overall median number of total appointments per year was 57.5 (IQR 19.0-115.5), equating to over one appointment per week. Adult patients had fewer total appointments (median 13.0, IQR 4.8-60.8) and consulted a smaller variety of health care professionals, with a median number of 4.5 types.

The median number of appointments over the past year was also higher (62.0, IQR 26.0-127.0) among caregivers impacted in their professional life compared to caregivers who experienced no such impact (44.0, IQR 18.0-102.0).

Overall, the most frequently consulted health care professionals were physiotherapists and pediatric neurologists or epileptologists (97/132, 73.5% and 96/132, 72.7%, respectively), followed by pediatricians (81/132, 61.4%). Half of the patients (69/132, 52.3%) consulted a speech therapist. Over the past year, patients with more frequent seizures consulted more frequently with pediatric neurologists or epileptologists. The consultation rates were as follows: 80% (43/54) for patients with 1-5 seizures per day, 78% (21/27) for patients with >5 seizures per day, 25% (3/12) for patients with a few seizures in a month, and 50% (4/8) for patients without seizures in the past month.

General practitioners were consulted more often in Europe (37/106, 34.9%) than elsewhere (3/26, 11.5%). Conversely, nutritionists, gastroenterologists, and geneticists were consulted 1.5 to 4 times more often outside Europe.

Patient age influenced the type of health care professionals consulted. Visits to the dentist in the past year were more frequently reported for older patients (16/20, 80% for patients aged ≥18 years and 20/23, 87% for patients aged 10-17 years) compared to younger patients (16/49, 33% for patients aged <5 years and 25/39, 64% for patients aged 5-9 years). Inversely, older patients had fewer appointments with ophthalmologists. They were visited by 80% (39/49) of those aged <5 years in the past year and only 10% (2/20) of those aged ≥18 years.

Of the 132 patients, 102 (77.3%) had been hospitalized and 89 (67.4%) visited the emergency department for CDD-related symptoms at least once in their lifetime (Figure S6 in Multimedia Appendix 3). The hospitalization rate was higher in Europe (86/106, 81.1%) than outside Europe (16/26, 62%), but the median length of stay during the past year was shorter in Europe (2.0 nights, IQR 0.0-11.5) than outside Europe (4.0 nights, IQR 0.8-10.8).

The percentage of hospitalized patients was consistent across age groups, but younger patients spent more nights at the hospital during the past year. Patients aged <5 years spent a median of 8.0 nights (IQR 2.0-30.0), while those aged 5-9 years spent a median of 3.5 nights (IQR 1.0-10.0). Older patients had fewer hospital stays, with a median of 0.0 for both age groups (those aged 10-17 years: IQR 0.0-2.2; those aged ≥18 years: IQR 0.0-2.5).

Both the number of hospital stays and the length of these stays tended to correlate positively with the frequency of seizures. Altogether 89% (24/27) of patients with >5 seizures a day were hospitalized at least once in their lifetime compared to 42% (5/12) of patients with only a few seizures per month. Patients experiencing >5 seizures per day had a median stay of 4.0 nights (IQR 0.8-13.2) at the hospital in the past year, while those with 1-5 seizures a day had a median stay of 6.0 nights (IQR 1.0-23.8). In contrast, patients with weekly seizures and those with monthly seizures spent a median of 0.0 nights (IQR 0.0-4.0 and IQR 0.0-4.5, respectively) at the hospital.

### Disease Burden for Patients With CDD

**Figure 6:**
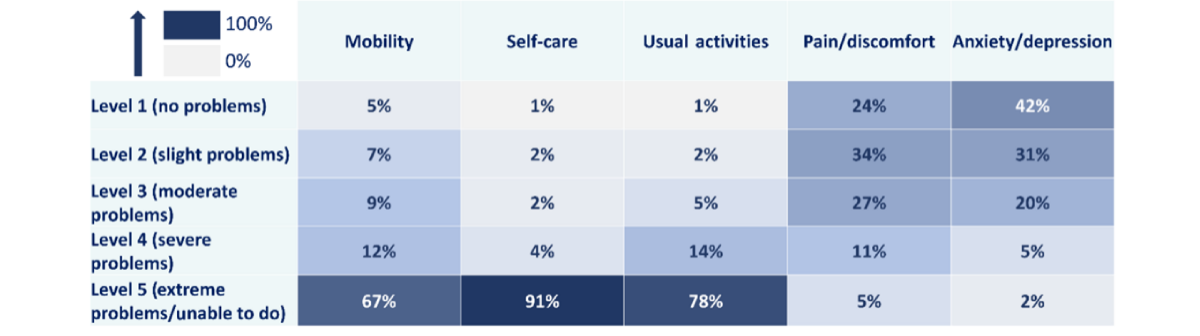
Caregiver perception of the severity of cyclin-dependent kinase-like 5 deficiency disorder on patients’ quality of life across the 5 EQ-5D-5L dimensions (N=132). For each dimension (mobility, self-care, usual activities, pain/discomfort, and anxiety/depression), the percentages of respondents are reported by the level of severity selected (1, “no problem” to 5, “extreme problems/unable to do”).

When completing the adapted version of the EQ-5D-5L, most of the caregivers declared that patients’ mobility (88/132, 66.7%), self-care (120/132, 90.9%), and everyday activities (103/132, 78.0%) were severely affected by CDD (level 5 “extreme problems/unable to do”) on the day of completion (Figure 6). For most of the patients, there was little to no pain or discomfort (77/132, 58.3%) and depression or anxiety (97/132, 73.5%) reported that day.

Caregivers mentioned consistent levels of severity for mobility, pain or discomfort, and depression or anxiety across all countries, but more European caregivers reported that patients with CDD were not able to wash or dress themselves (99/106, 93.4%) or perform usual activities (86/106, 81.1%) that day compared to non-European caregivers (21/26, 81% and 17/26, 65%, respectively) (Figure S7 in Multimedia Appendix 3).

Overall, severity appeared to be similar across most age groups, with the exception of mobility, which seemed to be less affected among older patients in this study. Among patients aged ≥18 years, only 35% (7/20) had a rating of level 5, indicating “extreme problems/unable to do” compared to 65% (15/23) of patients aged 10-17 years, 74% (29/39) of patients aged 5-9 years, and 76% (37/49) of patients aged <5 years.

The survey population showed a notably low health-related quality of life for patients with CDD, with an overall median EQ-5D-5L index value of 0.18 (IQR 0.11-0.32) on a scale ranging from 0 (a state comparable to being dead) to 1 (representing full health). The VAS results were different, with an overall median score of 56 out of 100 (IQR 39.0-71.0), suggesting a neutral stance on the health status of patients.

Median index values were slightly higher for patients aged <5 years (0.21, IQR 0.13-0.31) and patients aged ≥18 years (0.25, IQR 0.16-0.36) that for patients aged 5-17 years (patients aged 5-9 years: 0.13, IQR –0.09 to 0.27; patients aged 10-17 years: 0.17, IQR 0.08-0.35). Patients aged <10 years had lower VAS scores (patients aged <5 years: 52/100, IQR 35.0-75.0; patients aged 5-9 years: 50/100, IQR 39.0-62.5) compared to older patients (patients aged 10-17 years: 59/100, IQR 41.5-70.5), and patients aged ≥18 years had the highest median score (70/100, IQR 50.5-80.25), indicating a better health status as perceived by family caregivers.

### Caregiver Burden

#### Impact of CDD on Caregivers

In our sample, caring for a person with CDD had a substantial negative impact on all aspects of the caregiver’s life, with median ratings consistently exceeding 7 out of 10 across different domains (Figure S8 in Multimedia Appendix 3). The burdens on family, professional, and social life, and the levels of stress were mentioned particularly often. Family life and stress levels were rated with a median of 9 out of 10 (IQR 7.0-10.0 for both), and the social and professional spheres had a median rating of 9 out of 10 (IQR 6.0-10.0 for both).

Caregivers residing outside Europe reported a higher overall impact of CDD on their quality of life compared to their European counterparts (Figure S8 in Multimedia Appendix 3). The most striking difference was observed in the financial field, where European caregivers reported a median rating of 6 out of 10 (IQR 3.0-9.0), while caregivers from other regions of the world rated the financial impact at a median of 10 out of 10 (IQR 8.5-10.0), indicating a more severe financial strain outside Europe.

Three-quarters of caregivers (97/132, 73.5%) reported epilepsy or seizures as one of the most difficult symptoms to manage. This was followed by difficulty interpreting communication (51/132, 38.6%) and difficulty with walking or the inability to walk (45/132, 34.1%).

Caregivers who reported epilepsy as one of the most difficult symptoms to manage (n=97) also reported higher burdens on family, social, and professional life; quality of sleep; and levels of stress compared to those who did not mention this symptom (n=35; Table 3).

**Table 3:** Self-reported impact of cyclin-dependent kinase-like 5 deficiency disorder on caregivers’ family, social, and professional life; financial resources; sleep; stress; and overall quality of life, according to the level of epilepsy-related burden.

Aspects of life impacted by CDD^a,b^		Selected epilepsy/seizures as one of the hardest symptoms to manage (n=97)	Did not select epilepsy/seizures as one of the hardest symptoms to manage (n=35)
**Family life**			
	Mean (SD)	8.3 (2.5)	7.7 (2.5)
	Median (IQR)	10.0 (7.0-10.0)	8.0 (6.0-10.0)
**Social life**			
	Mean (SD)	8.0 (2.5)	7.5 (2.1)
	Median (IQR)	9.0 (7.0-10.0)	8.0 (6.0-10.0)
**Professional life**			
	Mean (SD)	8.1 (2.4)	6.8 (3.2)
	Median (IQR)	9.0 (7.0-10.0)	7.0 (5.0-10.0)
**Financial resources**			
	Mean (SD)	6.6 (3.2)	6.6 (3.5)
	Median (IQR)	7.0 (5.0-10.0)	7.0 (3.5-10.0)
**Quality of sleep**			
	Mean (SD)	7.6 (2.8)	7.2 (2.5)
	Median (IQR)	9.0 (6.0-10.0)	8.0 (6.0-10.0)
**Level of stress**			
	Mean (SD)	8.2 (2.3)	7.6 (2.2)
	Median (IQR)	9.0 (7.0-10.0)	7.0 (6.0-10.0)
**General quality of life**			
	Mean (SD)	7.9 (2.2)	7.2 (2.0)
	Median (IQR)	8.0 (7.0-10.0)	8.0 (5.0-9.0)

^a^CDD: cyclin-dependent kinase-like 5 deficiency disorder.

^b^Rated on a scale from 0 (no impact at all) to 10 (very high impact).

#### Out-of-Pocket Expenses

The majority of respondents (110/132, 83.3%) reported having out-of-pocket costs related to managing patients with CDD, with 44.7% (59/132) covering these expenses through household income (Figure S9 in Multimedia Appendix 3). However, 38.6% (51/132) found it challenging to meet these costs. While the number of caregivers reporting out-of-pocket expenses was consistent across all countries, more respondents from Europe were able to cover the costs (54/106, 50.9%) compared to non-Europeans (5/26, 19%).

Half of the respondents (55/110, 50.0%) reported incurring four or more different types of out-of-pocket expenses. The most common reason for expenses was alternative therapies, such as food supplements (66/110, 60.0%), followed by indirect costs (65/110, 59.1%) and nonpharmaceutical treatments (63/110, 57.3%).

**Figure 7:**
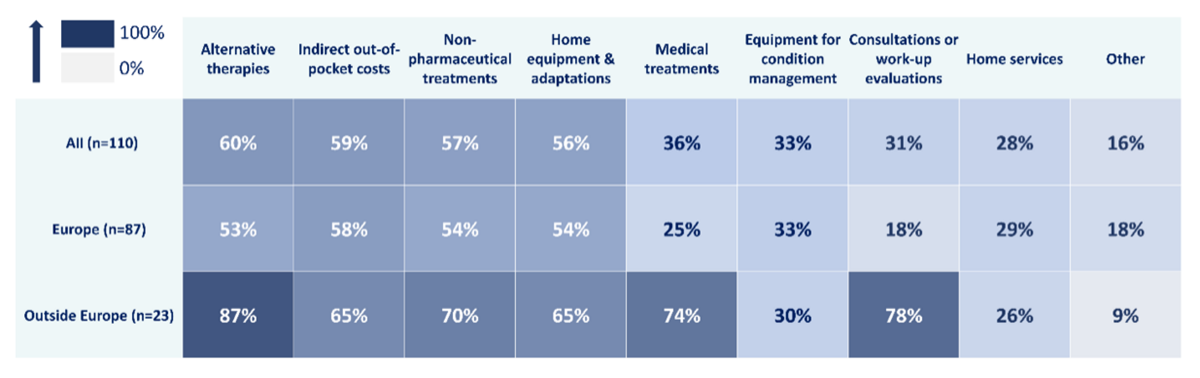
For caregivers who reported having out-of-pocket costs related to cyclin-dependent kinase-like 5 deficiency disorder management (n=110), the types of costs covered by themselves by region (Europe, n=87; outside Europe, n=23) are presented in terms of the percentage of respondents who selected each expense type.

The types of out-of-pocket costs varied greatly depending on the respondents’ region of residence (Figure 7). Across all regions, alternative therapy fees were the most frequently reported. However, outside Europe, fees for medical appointments or work-up evaluations (18/23, 78%) and medical treatments (17/23, 74%) were more frequently reported compared to Europe (16/87, 18% for consultations and 22/87, 25% for medical treatments).

Caregivers who reported professional life disruptions due to the disease faced greater difficulties in covering these costs (41/85, 48%) compared to those whose employment was not impacted (9/45, 20%).

#### Unmet Needs

When asked what kind of additional help they would like to have received, family caregivers mostly reported wishing for help with daily care and everyday activities (60/132, 45.5%) and improved medical care (57/132, 43.2%). Caregivers less frequently mentioned administrative and financial help (22/132, 16.7%), social and emotional support (20/132, 15.2%), and information on CDD (16/132, 12.1%).

## Discussion

### Main Results

In this study, we found that the perceived burden of CDD was substantial for patients and their caregivers. Using a tailored version of the EQ-5D-5L for this condition for the first time, we were able to better assess patients’ health-related quality of life. Caregiver burden was also reported to be significant, with CDD affecting multiple life aspects. Unmet needs were identified and appeared to be population-dependent, as variations between age groups and world regions were observed.

As previously reported for CDD, patients in this study had an early onset of seizures (median of 2.0 months), and most experienced daily seizures (66%), often of multiple types [4,9,14,27]. Variations regarding the proportion of patients experiencing different types of seizures, especially absence seizures, can be observed between this work and previous ones [14]. Identification of seizure types can be challenging for caregivers, as CDD seizures can present mixed motor and sometimes nonmotor features [14]. It is notably possible for caregivers to mistake epileptic spasms for hypermotor-tonic-spasm sequence seizures. Symptom load was high, with 57% of caregivers reporting eight or more symptoms. Over two-thirds of patients were hospitalized for CDD-related symptoms over the past year, and the proportion of hospitalized patients increased with the frequency of seizures. This aligns with previous findings that most hospitalizations of patients with CDD are related to seizures [29]. Almost all patients with seizures were on one or more ASMs (97%), and 82% of them discontinued at least one ASM. In previous work, the number of seizure-related hospital admissions increased with the number of ASMs used during the lifetime of patients [29]. All these findings may be an indication of the refractoriness of seizures, which is characteristic of CDD epilepsy [6,9,18,23,35].

The steady decline in caregiver satisfaction with the effectiveness of treatments in reducing seizures as time passed could be a further indication of potential treatment fatigue or habituation. Dissatisfaction with side effects, which usually consist of increased fatigue, irritability, and behavior disturbance, was also reported [18]. Polypharmacy, which was common in our study population, was previously associated with a higher likelihood of experiencing side effects and a lower quality of life for patients [18,28]. A ketogenic diet previously showed promising results for a short-term decrease in seizure frequency but poor long-term efficacy, which might explain the decrease in patients treated with this approach as they age [24].

The EQ-5D-5L is a standardized instrument used to quantitatively measure health-related quality of life, and it involves questions on health status [30,36]. In this study, most caregivers reported the highest severity in 3 out of the 5 dimensions measured: mobility, self-care, and everyday activities. Moreover, index values were low, with a median of 0.18, and were even negative for 17 respondents, indicating a perceived health state worse than death. This is consistent with previous research indicating that children with intellectual disabilities had lower EQ-5D scores than other populations [37]. Overall, these findings highlight the poor health-related quality of life of patients with CDD as perceived by their caregivers. The findings align with previous work using the Quality of Life Inventory Disability (QI-Disability), an instrument developed for children and adolescents with intellectual disability, which showed an association between more limited functional abilities and a poorer quality of life in patients with CDD [28,38]. These results provide some baseline data for comparison when using EQ-5D-5L as a standardized method for quantitatively assessing health-related quality of life in patients of all ages, enabling the analysis of various conditions and contributing factors. Furthermore, it may serve as a valuable complement to the QI-Disability scores in studies involving children and adolescents with CDD.

Family caregiver burden was reported as substantial in several aspects, both in this study and in previous work [25,26]. Most caregivers (64%) reported that their employment status was impacted by CDD. CDD care involved a wide range of health care professionals and was demanding and time-consuming, with the majority of patients requiring at least one appointment per week. Caregivers whose employment was impacted by CDD reported attending more appointments compared to those whose employment was not affected, highlighting the time-intensive nature of multidisciplinary care. Additionally, these caregivers faced greater financial challenges in covering costs related to the disease. This is a crucial issue, as a previous study has shown that financial strain is closely linked to a decline in parental well-being for CDD caregivers, emphasizing the importance of financial support for families [25].

Overall, caregivers reported substantial impacts of CDD on their family, social, and professional lives, as well as on their quality of sleep and stress levels. The burden of seizures was particularly significant for caregivers, with 73% identifying seizures as one of the most challenging symptoms to manage and reporting a greater impact on their own quality of life. This is consistent with findings that epilepsy, even in the absence of CDD or developmental delay, is associated with poorer family functioning [39].

These burdens and resulting unmet needs should be considered in the context of the profiles of both caregivers and patients, as noticeable differences were highlighted between world regions and age groups.

Caregivers outside Europe seemed to have more difficulties with access to resources and support, with only 19% of them reporting having access to a specialized CDD center compared to 51% of European respondents. Delay between the first seizure and diagnosis was also longer outside Europe than in Europe (26.5 months vs 11 months). This difference may be attributed to several factors, including varying levels of awareness of CDD, access to specialized medical centers, and the availability or reimbursement of genetic testing across different countries. CDD care varied by geographic location, with differences in the types of health care professionals consulted between European countries and countries outside Europe. The proportion of patients on ASMs and a ketogenic diet were similar across world regions. VNS was however reported exclusively among patients living in central, western, northern, and eastern Europe and was not reported among patients living outside Europe or in southern Europe. Major disparities in the availability of resources for neurological disorders exist across countries, with low-income countries being most severely disadvantaged [40]. These disparities could impact the level of care available for patients with CDD, potentially adversely impacting their quality of life [28].

Financial strain was also more severe for caregivers outside Europe, with a median rating of CDD impact on financial resources of 10 out of 10 compared to 6 out of 10 for European caregivers. Non-European caregivers reported greater difficulties in covering out-of-pocket fees related to medical treatments, medical appointments, or work-up evaluations, which were less frequently reported by Europeans. This may further highlight differences in health care systems and may suggest that European families benefit from better coverage and financial support, reducing the financial burden of CDD compared to families in other countries. Caregivers outside Europe were also more impacted in their professional life. All these difficulties had an impact on their quality of life, as caregivers outside Europe had higher median ratings of CDD impact on stress levels, social life, and general quality of life. All these findings are aligned with previous ones regarding the impacts of a family’s residential location and especially the presence of universal health care on financial well-being, which is often closely linked to overall well-being [25,41].

Many CDD symptoms are age-dependent [7,10,14]. In our survey, patients aged 5-9 years exhibited a higher number of symptoms, had more varied and frequent seizures, and were on more medications. This may be due to the course of the disease or individual variability, or because symptoms become more detectable by parents from the age of 5 years [10]. These patients also had poorer functional ability, as reflected by their EQ-5D-5L index values and VAS results. Adult patients in this study were reported to be notably less severely affected than children, exhibiting less frequent seizures, reduced visual impairment, and fewer walking difficulties. Moreover, they were reported to receive less comprehensive care, with fewer appointments and fewer different types of health care professionals involved. This suggests that the transition to adult care may pose challenges for patients with CDD, resulting in poorer care. Further evidence would be needed to better understand the biological and nonbiological factors that could impact the life and care of adult patients, as well as the rationale behind them being reported to be less affected than younger patients.

This highlights the need for an evolving, age-specific multidisciplinary care approach, as supported by previous work by Amin et al [3]. Finally, the age at diagnosis was reported to be higher for older patients, which may be explained by the relatively recent discovery of the disease in 2004 and the growing accessibility of genetic testing [2,14]. At present, patients are diagnosed at a younger age, and this trend is expected to continue, with the age at diagnosis anticipated to decrease and stabilize in the coming years.

### Strengths and Limitations

This study is one of the few studies illustrating the burden and impact of CDD on patients and caregivers with 132 respondents, mainly from western parts of Europe, Latin America, and Turkey. Clinical features of patients reported by caregivers were consistent with the epidemiological and clinical characteristics reported in the literature, including female predominance, early onset of seizures, impaired development, limited communication and gross motor skills, high seizure frequency, and multiple seizure types [5,10,14,42,43]. Treatments reported aligned with those commonly described in the literature [18,23,24]. Despite self-reporting by caregivers, the consistency of reported medical data with the literature verifies the representativeness of our study’s sample.

To measure health-related quality of life, we used an adapted and validated version of a standardized tool, the EQ-5D-5L, as described in the methods. This tool has been used in several studies and demonstrated excellent psychometric properties across a broad range of populations [44]. However, dimension scores for conditions associated with intellectual disability are usually much lower than for other chronic health conditions but map to other measures of the dimension (eg, mobility and self-care), highlighting that scores reflect relevant impairments and functions [28,36,37]. Different value sets were used to obtain the EQ-5D-5L index values of countries, complicating cross-country comparisons, as differences may result from the value sets rather than from the respondents’ answers [45] (Multimedia Appendix 2).

In this study, comparisons between European respondents (n=106) and those from outside Europe (n=26; mostly from Southern America and Turkey) were unbalanced, with a higher number of participants from Europe and especially from Western Europe. Patients’ medical characteristics varied between countries, and these disparities may have influenced the cross-country analysis, limiting the generalizability of the results. Participation in the survey was voluntary, with most participants recruited via support organizations and the Carenity platform. This may have led to an overrepresentation of caregivers for patients with severe symptoms, while isolated caregivers or those without internet access were underrepresented. This also may have impacted the geographic distribution of the respondents, as they were recruited from locations where active support organizations exist. Additionally, women and younger adults are often overrepresented in online communities, potentially influencing the sample composition [46].

If a respondent stopped and closed the questionnaire during completion, it was technically not possible for them to resume where they left off and they had to start over. This limitation may have contributed to a reduction in the number of fully completed and analyzable surveys.

The same team member conducted both the quality check and analysis, which may have introduced slight bias. However, another analyst, independent of the project, reviewed both the quality check and analysis results.

### Future Directions and Implications

Our findings highlight the critical need for new effective ASMs as part of CDD treatment, benefiting not only patients but also their families, whose well-being is closely tied to the management of the condition. The use of a tailored version of the EQ-5D-5L to quantify health status in this survey generated values that could potentially serve as reference points for future studies to evaluate the impact of treatments, other therapies, practices, or other factors. As the EQ-5D-5L is a measure of health-related quality of life and is therefore influenced by impairments, it could benefit from being complemented by a measure of quality of life such as the QI-Disability score.

Solutions regarding support and access to resources must be adjusted depending on the family location, as considerable geographical discrepancies exist. The care provided for patients with CDD should evolve as they age, be multidisciplinary, and be adapted to the clinical features exhibited by the patient, as significant individual variations exist for this disorder [10].

### Conclusion

Our results reveal the significant burden of CDD on both patients and caregivers, affecting caregivers’ quality of life, including financial resources, sleep, stress levels, and professional and social aspects. Seizures in particular have a profound impact on caregivers’ quality of life, underscoring the need to develop efficient and long-lasting ASMs. Furthermore, the study highlights geographical discrepancies regarding access to specialized medical centers, diagnosis delays, health-related cost coverage, and financial resources. These findings suggest that disease management and support should be tailored to the specific needs and health care systems of each region.

For the first time, the health-related quality of life of patients with CDD was measured by caregivers using a tailored version of the standardized and validated EQ-5D-5L tool, which provides a valuable baseline for future research.

The observed differences between age groups emphasize the need for multidisciplinary care for all patients, including adult patients with CDD, who appear to receive less comprehensive care. Further research is needed on caregivers outside Europe, children aged 5-9 years, and adult patients.

## Appendix

Multimedia Appendix 1 Survey questionnaire.

Multimedia Appendix 2 Value sets used for index value calculation by country.

Multimedia Appendix 3 Additional figures related to geographic location, age, and seizure frequency.

Multimedia Appendix 4 CHERRIES checklist.

## Abbreviations

ASM: antiseizure medication
CDD: cyclin-dependent kinase-like 5 deficiency disorder
CDKL5: cyclin-dependent kinase-like 5
QI-Disability: Quality of Life Inventory-Disability
VAS: visual analog scale
VNS: vagus nerve stimulation

## Data Availability

The datasets generated or analyzed during this study are not publicly available due to personal data protection policies but are available from the corresponding author upon reasonable request.
