# Supplementary material for: Caregivers’ Perceptions of Clinical Symptoms, Disease Management, and Quality of Life Impact in Cases of Cyclin-Dependent Kinase-Like 5 Deficiency Disorder: Cross-Sectional Online Survey
Source: JMIR Form Res. 2025 Jun 10;9:e72489. doi: 10.2196/72489 (PMC12188142; doi:10.2196/72489)
Supplement: Multimedia Appendix 1 [file formative_v9i1e72489_app1.docx]

# Survey questionnaire

**Burden and unmet needs linked to CDKL5 Deficiency Disorder, and impact on quality of life**

**Introduction message for Carenity participants**

Hi,

This is a survey dedicated to caregivers of persons living with **CDKL5 deficiency disorder (CDD).**

The aim of this survey is to assess and better understand the burden of this disease and the impact on the quality of life of persons with CDD and their caregivers.

It is estimated that it will take approximately 25 minutes to complete the full questionnaire.

Data collected through this survey will be handled and shared anonymously with Orion Corporation, a Finnish pharmaceutical company, that is sponsoring this survey. The data will be processed according to General Data Protection Regulation and not transferred outside Europe.

The results of this survey may also be used externally in scientific publications or communications during scientific congresses. In addition, the results will be communicated through International CDKL5 Alliance and will hopefully benefit the entire community.

*According to current legislation, you have the right to access, modify and oppose to the processing of your data at any time.*

*To learn more about the way your data will be used and about the terms of your rights, please read the Information and Consent Notice.*

*Your participation in the survey is voluntary. By clicking on the “Start” button, you confirm:*
*1. your willingness to participate in the survey; and*
*2. that you give your explicit consent to have your personal data collected and processed in the way described in our* [*Information and Consent Notice*](https://cdn-member.carenity.co.uk/docs/20221109-Orion-CDD-ICF-Carenity-UK-1667984824.pdf)*.*

Thank you in advance for your participation.

The Carenity team

1. **SOCIODEMOGRAPHIC AND MEDICAL CHARACTERISTICS**
2. **Sociodemographic profile**
3. **Do you take care of a person with CDKL5 Deficiency Disorder (CDD)?**

*Please select only one answer.*

- Yes, I am a caregiver of a person with CDD
- I was a caregiver of a person with CDD. The person of whom I took care has passed away but I want to answer the questionnaire
- Yes, a person close to me is affected by CDD but I am not the main caregiver 🡪 Please specify: [Free field]
- No, I don’t know anyone with CDD **[END SURVEY]**

**[This question will be asked only to caregivers who ticked they lost the person they were taking care of]**

1. **When did the person you were taking care of pass away?**

*Please select a year in the calendar by clicking the field below.*

*(YYYY)*

**[This description will only be displayed to caregivers who ticked they lost the person they were taking care of]**

*You will now see several questions on the sociodemographic and medical profile of the person you cared for. Please answer them with the information before the person passed away.*

*The questionnaire is written in the present tense.*

1. **Are you the person’s legal guardian?**

*In this survey, you may share personal health information about a child affected by CDD. Please note that you can only do so if you are the child's legal guardian. If you are not, you will not be able to answer the questionnaire.*

*Please select only one answer.*

- Yes
- No **[END SURVEY]**

1. **What is your relationship with the person for whom you provide care?**

*Please select only one answer.*

- I am the person’s mother/father
- I am the person’s grandmother/grandfather
- I am the person’s sibling
- I am the person’s uncle/aunt
- I am a professional carer for the person
- I am the person’s primary guardian (but not related)
- Other 🡪 Please specify: [Free field]

1. **Your country of residence:**

*Please select only one answer.*

- Austria
- Belgium
- Denmark
- England
- Finland
- France
- Germany
- Italy
- The Netherlands
- Northern Ireland
- Norway
- Poland
- Portugal
- Republic of Ireland/ Southern Ireland
- Scotland
- Spain
- Sweden
- Switzerland
- Wales
- Other 🡪 Please specify: [Free field]

1. **Your gender:**

*Please select only one answer.*

- Female
- Male
- Prefer not to answer

1. **Gender of the person for whom you provide care:**

*Please select only one answer.*

- Female
- Male

1. **Your year of birth:**

*Please select a year in the calendar by clicking the field below.*

*(YYYY)* [if <18 years old, end of the survey]

1. **The age of the person for whom you provide care:**

*Please fill in the year and month of birth of the person.*

*(Numeric fields in year and month)*

1. **What is your employment status?**

*Please select only one answer.*

- I work full time
- I work part time because the person for whom I provide care has CDD
- I work part time because of other reasons (not linked to the person’s CDD)
- I do not work because the person for whom I provide care has CDD
- I do not work because of other reasons (not linked to the person’s CDD, ex: retired, student, etc.)
- Other 🡪 *Please specify:* [Free field]

1. **Where does the person affected by CDD currently live?**

*Please select only one answer.*

- In the family home
- In a community home
- In a specialized institution
- Other 🡪 *Please specify:* [Free field]

1. **Does the person have access to specialized healthcare centres, for example clinics or hospitals, with expertise in CDD?**

*Specialized healthcare centres are CDKL5 Centres of Excellence, or clinics, or hospitals, specialized in comprehensive management of rare genetic epilepsies.*

*Please select only one answer.*

- Yes
- No
- Other 🡪 *Please specify:* [Free field]

1. **Medical profile & patient pathway**
2. **At what age did the person start having seizures or other symptoms?**

*If you are not certain about the exact age, please give your best estimation.*

*Please fill in the age in years and months in the fields below.*

*(Numeric fields in years and months)*

1. **At what age did the person first see a doctor for their symptoms?**

*If you are not certain about the exact age, please give your best estimation.*

*Please fill in the age in years and months in the fields below.*

*.*

*(Numeric fields in years and months)*

**[Questions 15 and 15bis will be displayed on the same page.]**

1. **At what age was the person diagnosed with CDKL5 Deficiency Disorder (CDD)?**

*If you are not certain about the exact age, please give your best estimation.*

*Please fill in the age in years and months in the fields below.*

*(Numeric fields in years and months)*

**Q15bis. Did the person have a genetic test to confirm diagnosis?**

- *Yes*
- *No* **[TICKED: END SURVEY]**

1. **Which of the following has the person experienced over the past year?**

*(Multiple answers – randomized items, minimum 1 item)*

*Please select all that apply.*

- Epilepsy/seizures
- Difficulties with walking or unable to walk
- Scoliosis or kyphosis (curvature of the spine)
- Movement disorders (e.g., involuntary, erratic movements, etc.)
- Stereotypies (these are purposeless and repetitive hand movements such as clapping, hand licking or sucking…)
- Limited hand function
- Visual impairment
- Limited communication skills and/or speech
- Behaviour disturbances
- Sleep problems
- Gastrointestinal and feeding problems
- Cardiac issues
- Respiratory problems
- Other 🡪 *Please specify:* [Free field]
- The person did not experience any symptoms **[EXCLUSIVE]**

**[This question will be asked only to patients who ticked “Epilepsy/seizures” in Q16]**

1. **What type(s) of seizures did the person experience over the past year?**

*(Randomized answers)*

*Please select all that apply.*

- Major motor seizures (Atonic/drop, clonic, tonic, and/or generalized tonic-clonic)
- Hypermotor-tonic-spasms sequence (HTSS)
- Epileptic spasms
- Myoclonic seizures
- Absence seizures
- Other 🡪 *Please specify:* [Free field]
- I am not sure of all the types of seizures the person I take care of is having
- I do not remember **[EXCLUSIVE]**

*Epileptic spasms are very brief seizures characterized by sudden flexion and/or extension, often in the arms.*

*Myoclonic seizures are characterized by one or more spasms that occur in the whole body or in one arm or leg, during which the person remains conscious.*

*Tonic seizures are manifested by a sudden fall, loss of consciousness, sustained tonic contraction of all muscles, apnoea, airway mucus hypersecretion and salivary hypersecretion. Sometimes the affected person may bite the side of their tongue.*

*Tonic-clonic seizures occur in two phases: the tonic phase and the clonic phase. Loss of consciousness occurs during the tonic phase. The person becomes stiff and has generalized muscle contractions all over the body for about 20 seconds. The seizure may last a few minutes. At the end of the seizure, the person relaxes and falls asleep.*

*Atonic/drop seizures consist in a sudden loss of muscle strength, that may cause the person to fall to the ground, or their head to drop.*

*Hypermotor-tonic-spasms sequence (HTSS) is a distinctive seizure type found in persons with CDKL5 deficiency disorder. It consists of an initial hypermotor phase involving limbs and trunk movements (kicking, rocking…), followed by a tonic phase (sustained tonic contraction of all muscles), and a final spastic phase involving sudden, involuntary extension of the limbs.*

*In absence seizures, the person may appear to be staring into space and/or have jerking or twitching muscles.*

**[This question will be asked only to patients who ticked “Epilepsy/seizures” in Q16]**

1. **How often has the person experienced seizures over the past month?**

*Please select only one answer.*

- More than 5 seizures a day
- 1 to 5 seizures a day
- Weekly
- Only a few in the month
- The person did not experience any seizures

**[This question will be asked only to patients who ticked “Epilepsy/seizures” in Q16]**

1. **Is the person receiving treatment for epileptic seizures?**

*Please select* ***all that apply****.*

- Yes, the person takes antiseizure medications 🡪 *How many regular antiseizure medications is the person currently on?* [Numeric field] / I don’t know
- Yes, a ketogenic diet
- Yes, the person has vagus nerve stimulation
- No
- I don’t know

1. **How many antiseizure medications has the person with CDD stopped using since the diagnosis?**

*Please answer this question by indicating how many medications have previously been tried but are no longer used. If you are not sure, please give us your best estimation.*

*[Numeric field]*

- I don’t know

**[This question will be asked only to patients who ticked “Epilepsy/seizures” in Q16]**

1. **How frequently did the person use rescue medications over the past year?**

*Rescue medications are medications that are given only in the event of a seizure, in order to stop it quickly and to prevent emergency situations.*

*Please select only one answer.*

- Once or more a day
- Once a week or several times a month
- Once a month or less
- A few times a year
- Never
- I don’t know

**[This question will be asked only to patients who ticked “Epilepsy/seizures” in Q16]**

1. **Over the past year, how many times could you have used rescue medication for the person you take care of but chose not to?**

*If you are not certain about the exact number, please give your best estimation.*

*Please fill in the number of times in the field below.*

*(Numeric fields)*

1. **With which healthcare professionals did the person have an appointment over the past year?**

*Please select all that apply.*

- Paediatrician 🡪 *Please give us your best estimation of the number of appointments with* ***this healthcare professional*** *over the past year:* [Free field] appointments
- General Practitioner 🡪 *Please give us your best estimation of the number of appointments with* ***this healthcare professional*** *over the past year:* [Free field] appointments
- Paediatric neurologist or epileptologist 🡪 *Please give us your best estimation of the number of appointments with* ***this healthcare professional*** *over the past year:* [Free field] appointments
- Neurologist or epileptologist🡪 *Please give us your best estimation of the number of appointments with* ***this healthcare professional*** *over the past year:* [Free field] appointments
- Geneticist🡪 *Please give us your best estimation of the number of appointments with* ***this healthcare professional*** *over the past year:* [Free field] appointments
- Ophthalmologist🡪 *Please give us your best estimation of the number of appointments with* ***this healthcare professional*** *over the past year:* [Free field] appointments
- Respiratory physician🡪 *Please give us your best estimation of the number of appointments with* ***this healthcare professional*** *over the past year:* [Free field] appointments
- Gastroenterologist🡪 *Please give us your best estimation of the number of appointments with* ***this healthcare professional*** *over the past year:* [Free field] appointments
- Cardiologist🡪 *Please give us your best estimation of the number of appointments with* ***this healthcare professional*** *over the past year:* [Free field] appointments
- Dentist🡪 *Please give us your best estimation of the number of appointments with* ***this healthcare professional*** *over the past year:* [Free field] appointments
- Nurse (epilepsy/community/district) 🡪 *Please give us your best estimation of the number of appointments with* ***this healthcare professional*** *over the past year:* [Free field] appointments
- Mental health professional (e.g., psychiatrist, community mental health team, etc.) 🡪 *Please give us your best estimation of the number of appointments with* ***this healthcare professional*** *over the past year:* [Free field] appointments
- Neuropsychologist🡪 *Please give us your best estimation of the number of appointments with* ***this healthcare professional*** *over the past year:* [Free field] appointments
- Occupational therapist 🡪 *Please give us your best estimation of the number of appointments with* ***this healthcare professional*** *over the past year:* [Free field] appointments
- Speech and language therapist🡪 *Please give us your best estimation of the number of appointments with* ***this healthcare professional*** *over the past year:* [Free field] appointments
- Physiotherapist🡪 *Please give us your best estimation of the number of appointments with* ***this healthcare professional*** *over the past year:* [Free field] appointments
- Nutritionist🡪 *Please give us your best estimation of the number of appointments with* ***this healthcare professional*** *over the past year:* [Free field] appointments
- Orthopaedics🡪 *Please give us your best estimation of the number of appointments with* ***this healthcare professional*** *over the past year:* [Free field] appointments
- Other 🡪 *Please specify:* [Free field]

1. **Has the person ever been hospitalized for CDD-related symptoms?**

*Please select only one answer.*

- Yes 🡪 *Please give your best estimate of the number of nights that the person with CDD stayed in hospital over the past year.* [Numeric field] nights a year
- No

1. **Has the person ever gone to an emergency room for CDD-related symptoms?**

*Please select only one answer.*

- Yes 🡪 *Please give your best estimate of the number of times that the person with CDD has needed an emergency room over the past year.* [Numeric field] times a year
- No

1. **Burden of the disease**

**PRO: Version Proxy1 of the EQ5D**

*Please select the ONE box that you think best describes the person’s health TODAY.*

*You should not answer on behalf of the person, but rather rate the person’s health as you see it.*

1. *MOBILITY*

- No problems in walking about
- Slight problems in walking about
- Moderate problems in walking about
- Severe problems in walking about
- Unable to walk about

1. *SELF-CARE*

- No problems washing or dressing him/herself
- Slight problems washing or dressing him/herself
- Moderate problems washing or dressing him/herself
- Severe problems washing or dressing him/herself
- Unable to wash or dress him/herself

1. *USUAL ACTIVITIES* (e.g. work, study, housework, family or leisure activities)

- No problems doing his/her usual activities
- Slight problems doing his/her usual activities
- Moderate problems doing his/her usual activities
- Severe problems doing his/her usual activities
- Unable to do his/her usual activities

1. *PAIN / DISCOMFORT*

- No pain or discomfort
- Slight pain or discomfort
- Moderate pain or discomfort
- Severe pain or discomfort
- Extreme pain or discomfort

1. *ANXIETY / DEPRESSION*

- Not anxious or depressed
- Slightly anxious or depressed
- Moderately anxious or depressed
- Severely anxious or depressed
- Extremely anxious or depressed
- We would like to know how good or bad you think the person’s health is TODAY.
- You will see a scale numbered from 0 to 100.
- 100 means the best health you can imagine.0 means the worst health you can imagine.
- Please indicate on the scale how you think the person’s health is TODAY.

| The Person’s Health Today | The worst health you can imagine | ------------O------------ | The best health you can imagine |
| --- | --- | --- | --- |

**[This description will only be displayed to caregivers who ticked they lost the person they cared for]**

*You will now see several questions on the burden of the disease. Please answer them with the information before the person passed away.*

1. **Which of the following are the most difficult for you to manage as a carer?**

*(Randomized answers)*

*Please select up to 3 symptoms.*

- Epilepsy/seizures
- Difficulties with walking or unable to walk
- Scoliosis or kyphosis (curvature of the spine)
- Movement disorders (e.g., involuntary, erratic movements, etc.)
- Stereotypies (these are purposeless and repetitive hand movements such as clapping, hand licking or sucking…)
- Limited hand function
- Visual impairment
- Difficulty interpreting communication
- Behaviour disturbances
- Sleep problems
- Gastrointestinal and feeding problems
- Cardiac issues
- Respiratory problems
- Other 🡪 *Please specify:* [Free field]
- The person does not experience any symptom **[EXCLUSIVE]**

1. **How would you rate the impact of CDD on the following aspects of your life (as a carer)?**

*Please drag the slider to the desired position (left end = no impact at all, right end = very high impact).*

| Family life | No impact at all | ------------O------------ | Very high impact |
| --- | --- | --- | --- |
| Social life | No impact at all | ------------O------------ | Very high impact |
| Professional life | No impact at all | ------------O------------ | Very high impact |
| Financial resources | No impact at all | ------------O------------ | Very high impact |
| Quality of sleep | No impact at all | ------------O------------ | Very high impact |
| Level of stress | No impact at all | ------------O------------ | Very high impact |
| **General quality of life** | No impact at all | ------------O------------ | Very high impact |

**[This question will be asked only to caregivers from the patient’s family]**

1. **Do you have any out-of-pocket costs from managing the disease?**

*Please select only one answer.*

- Yes, and my household finds it difficult to cover these costs
- Yes, but my household revenues allow us to cover these costs
- No

**[This question will be asked only to respondents who have out-of-pocket fees]**

1. **Which types of out-of-pocket costs do you have to cover?**

*(Randomized answers)*

*Please select all that apply.*

- Medical treatments
- Non-pharmaceutical treatments (e.g., physiotherapy, etc.)
- Linked to alternative therapies (e.g., food supplements, etc.)
- Linked to consultations or work-up evaluations (e.g., EEG, etc.)
- Linked to home services (e.g., nurse/carer assisting the person for daily tasks, doing activities with the person, etc.)
- Linked to home equipment and adaptations (i.e., medical bed, reducing household safety hazards, etc.)
- Linked to equipment for the management of the condition
- Indirect out-of-pocket costs (e.g., time off for medical appointments, etc.)
- Other 🡪 *Please specify:* [Free field]

1. **Treatment and support**

**[This question will be asked only to patients who ticked “Yes” in Q19]**

1. **How satisfied are you regarding the person’s current treatment for epileptic seizures?**

*Please drag the slider to the desired position (left end = not satisfied at all, right end = very satisfied).*

| Reducing seizures (frequency and/or severity) | Not satisfied at all | ------------O------------ | Very satisfied |
| --- | --- | --- | --- |
| Side effects | Not satisfied at all | ------------O------------ | Very satisfied |
| Method and burden of administration | Not satisfied at all | ------------O------------ | Very satisfied |

1. **What would you expect from a future treatment for epileptic seizures?**

*(Ranking question – randomized items, minimum 1 item)*

*Please select all the aspects that are the important to you for seizures treatment, in the order of their importance, so the first aspect you select is the most important for you, and so on.*

*If you want to change the order, you can unselect an item by clicking on it again.*

*1: this aspect is the most important for me, 2: this aspect is the second most important for me, etc.*

- That it decreases the number of seizures
- That it increases the time between seizures
- That it decreases the severity of seizures
- That it has a positive impact on cognition and neurodevelopment
- That it has fewer side effects
- That it is less burdensome to take/administer
- That it is cheaper
- Other 🡪 *Please specify:* [Free field]

1. **Do you know if one or more CDD patient support organizations exist in your country?**

*Please select only one answer.*

- Yes 🡪 Do you belong to one of them? If so, please give the name of the association(s)? [Free field]
- No 🡪 Would you be willing to get involved with one of them? [Free field]

1. **When thinking about care so far, what additional help would you or your family have liked to have received (or would have liked the person to receive)?**

*Please detail your answer.*

| *Free field* |
| --- |

1. **Where did you hear about the survey?**

*Please select all that apply*

- CDKL5 Alliance
- Local patient association
- Epicare
- Treating physician
- Nurse or other relevant
- Social media
- Carenity
- Other 🡪 *Please specify:* [Free field]

**Closing and thank you messages.**

This is a Multimedia Appendix to a full manuscript published in the J Med Internet Res. For full copyright and citation information see http://dx.doi.org/10.2196/jmir.xxxx
