# Supplementary material for: Caregivers’ Perceptions of Clinical Symptoms, Disease Management, and Quality of Life Impact in Cases of Cyclin-Dependent Kinase-Like 5 Deficiency Disorder: Cross-Sectional Online Survey
Source: JMIR Form Res. 2025 Jun 10;9:e72489. doi: 10.2196/72489 (PMC12188142; doi:10.2196/72489)
Supplement: Multimedia Appendix 2 [file formative_v9i1e72489_app2.docx]

**Multimedia Appendix 2.** Value sets used for index value calculation by country.

| **Country** | **Value set used** |
| --- | --- |
| Argentina | **Uruguay** |
| Australia | Australia |
| Austria | **Germany** |
| Belgium | Belgium |
| Bolivia | **Peru** |
| Chile | **Peru** |
| Colombia | **Peru** |
| France | France |
| Germany | Germany |
| Ireland | Ireland |
| Italy | Italy |
| Luxembourg | **France** |
| Mexico | Mexico |
| Montenegro | **Slovenia** |
| Netherlands | Netherlands |
| Peru | Peru |
| Poland | Poland |
| Portugal | Portugal |
| Slovakia | **Slovenia** |
| Spain | Spain |
| Sweden | Sweden |
| Switzerland | **France** |
| Turkey | **Iran** |
| UK | UK |
| Uruguay | Uruguay |
| USA | USA |

In bold are the countries for which no value set was available and for which the value set from a neighboring country was used.

This is a Multimedia Appendix to a full manuscript published in the J Med Internet Res. For full copyright and citation information see http://dx.doi.org/10.2196/jmir.xxxx
