# Supplementary material for: Caregivers’ Perceptions of Clinical Symptoms, Disease Management, and Quality of Life Impact in Cases of Cyclin-Dependent Kinase-Like 5 Deficiency Disorder: Cross-Sectional Online Survey
Source: JMIR Form Res. 2025 Jun 10;9:e72489. doi: 10.2196/72489 (PMC12188142; doi:10.2196/72489)
Supplement: Multimedia Appendix 3 [file formative_v9i1e72489_app3.docx]

**Multimedia Appendix 3.** Additional figures related to geographic location, age, and seizure frequency.

**Figure S1: Age at first seizure, age at diagnosis and delay between first seizures/symptoms and diagnosis by geographic location**
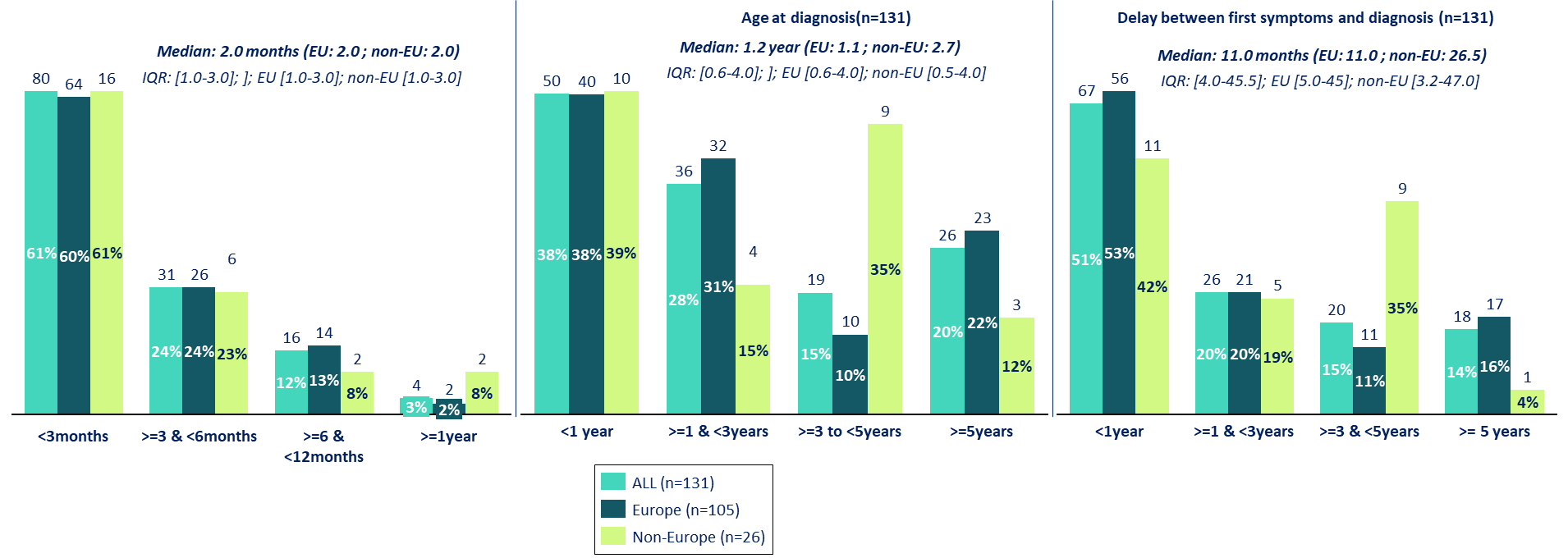


**Figure S2: Number of daily antiseizure medications (ASMs) by frequency of seizures experienced over the past month** **by patients with CDD. Patients who experienced seizures and were treated with ASM were categorized into subgroups according to seizure frequency over the past month: no seizure (n=7), a few seizures in the month (n=10), weekly seizures (n=20), one to five seizure a day (n=54), more than five seizures a day (n=26)**


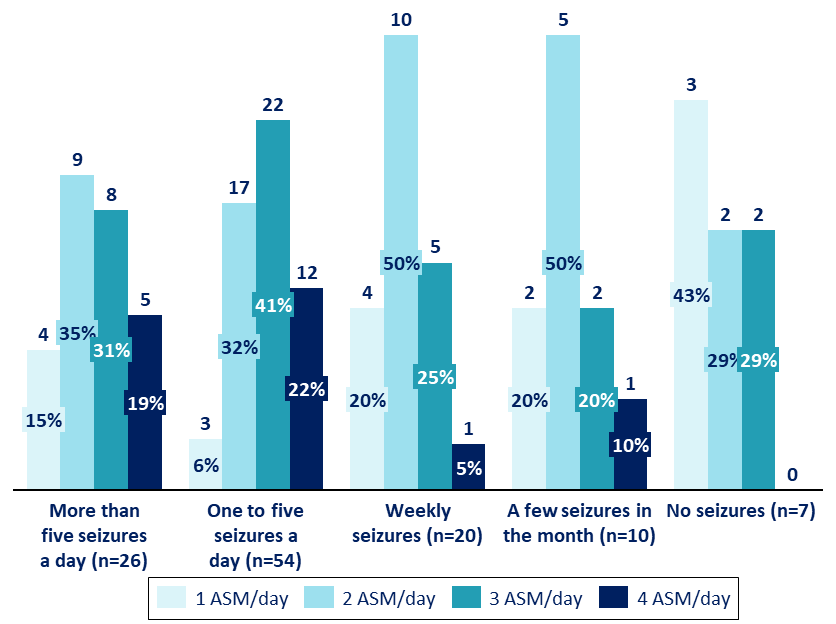


**Figure S3: Treatment for epileptic seizures by geographic location**
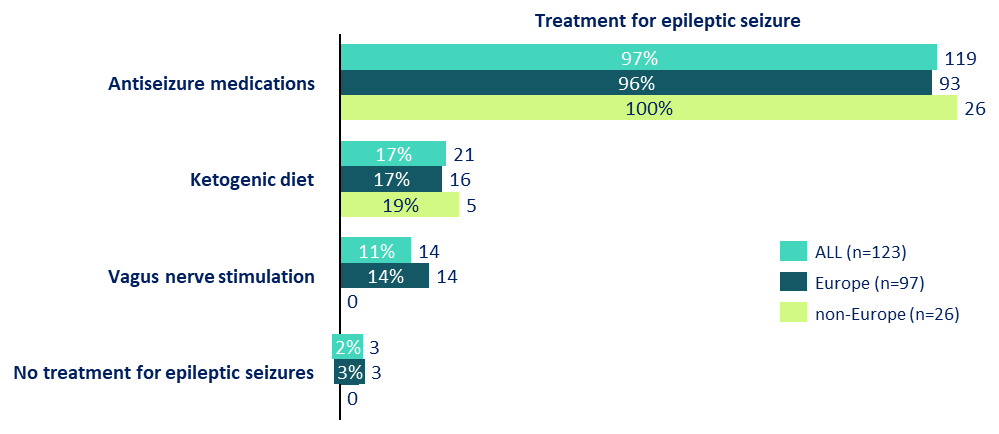


**Figure S4: Frequency of rescue medication use and opportunities over the past year by age group at inclusion in patients with CDD. Patients who experienced seizures were categorized into age subgroups : <5 y/o (n=47), >=5 & 10 y/o (n=35), >=10 & <18 y/o (n=21), >=18 y/o (n=19)**


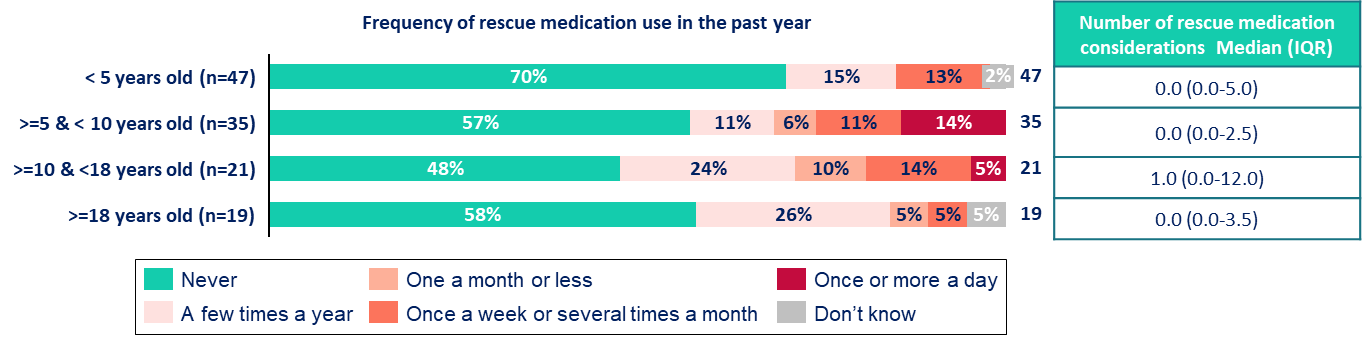


**Figure S5: Appointments with healthcare professionals over the past year by geographic location**


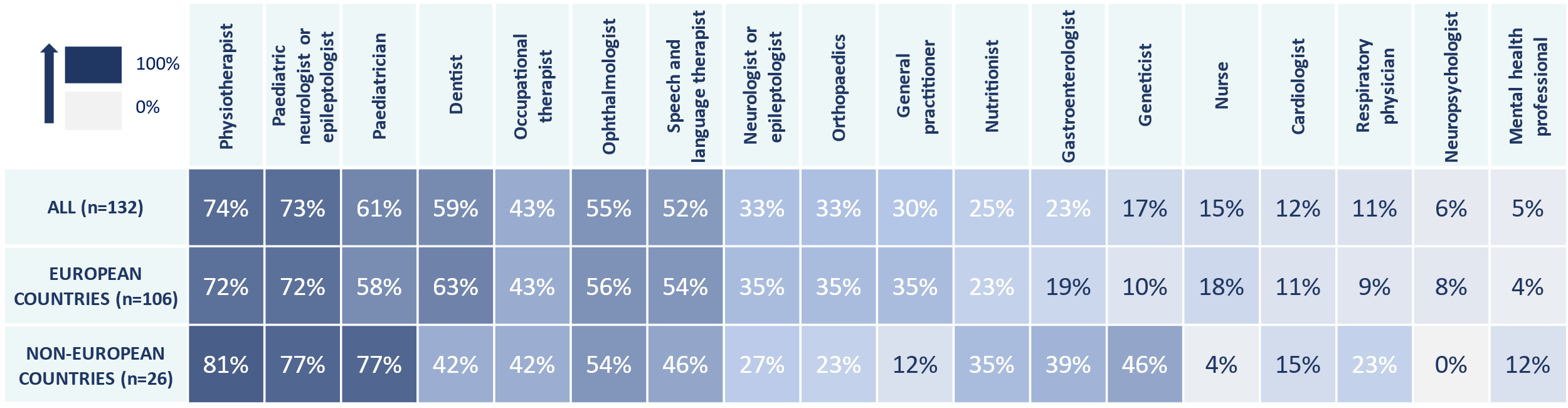


**Figure S6: Hospitalization for CDD-related symptoms over the past year by geographic location**
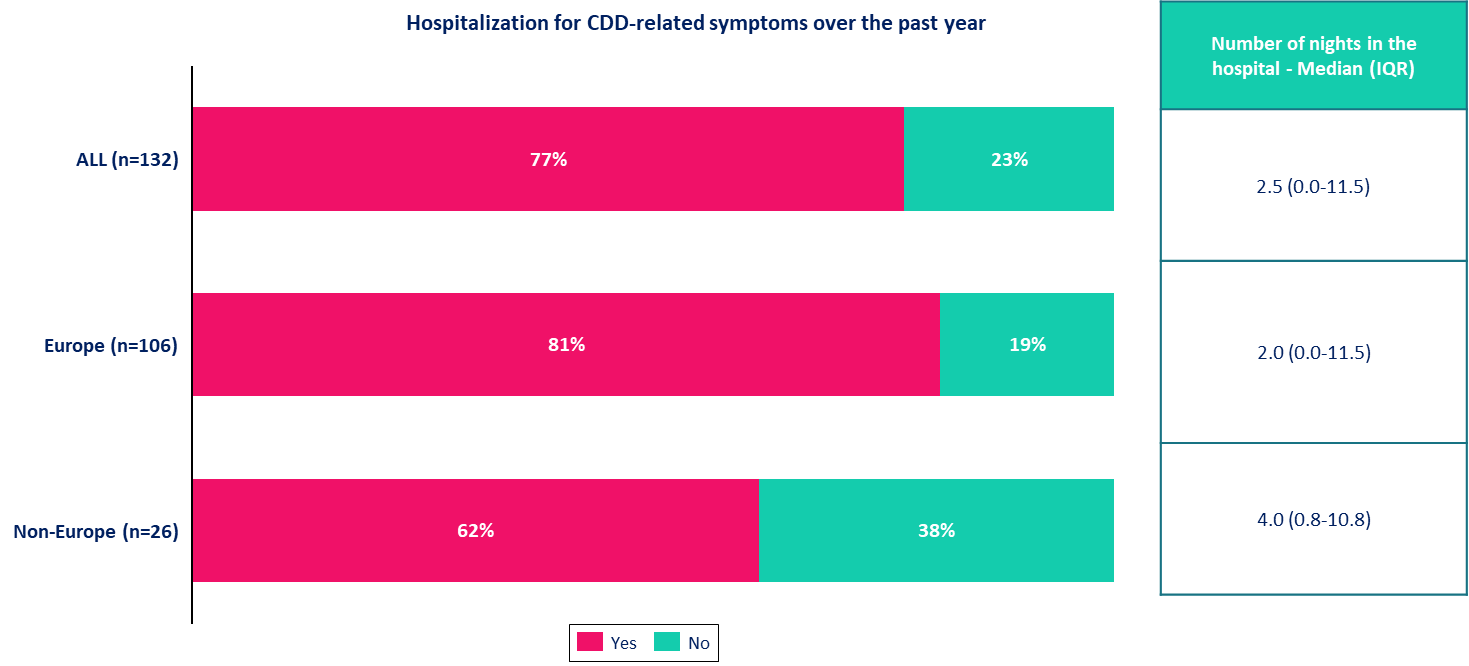


**Figure S7: EQ-5D-5L proportions reported by dimension and level by geographic location**


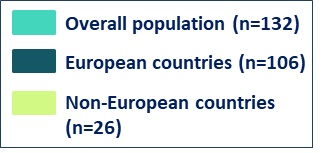

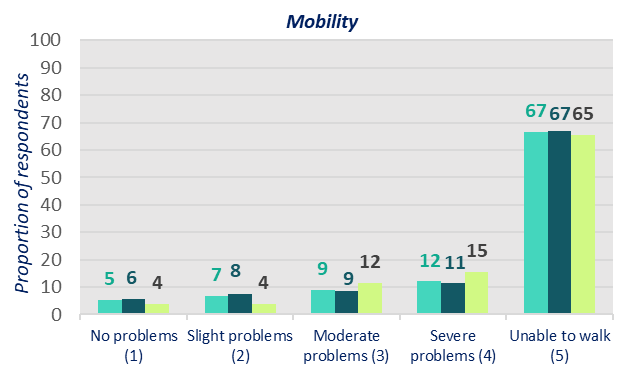


**
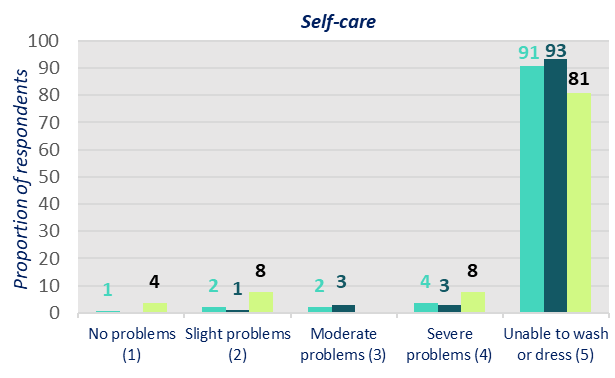
**
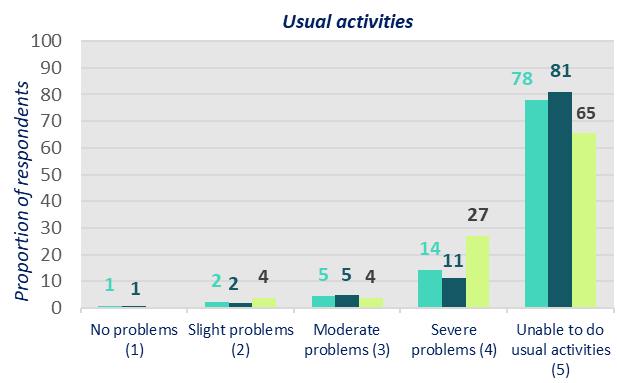

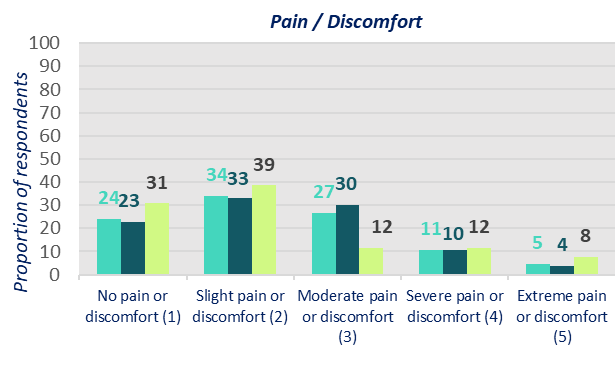
**
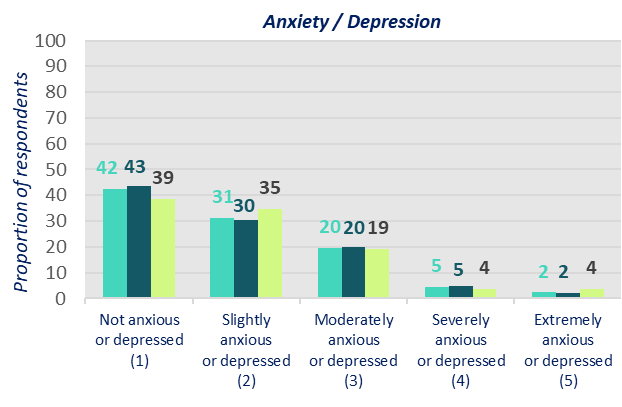
**

**Figure S8: Median rating of caregivers’ reported Impact of CDD on their family, social and professional life, financial resources, quality of sleep, level of stress and general quality of life**
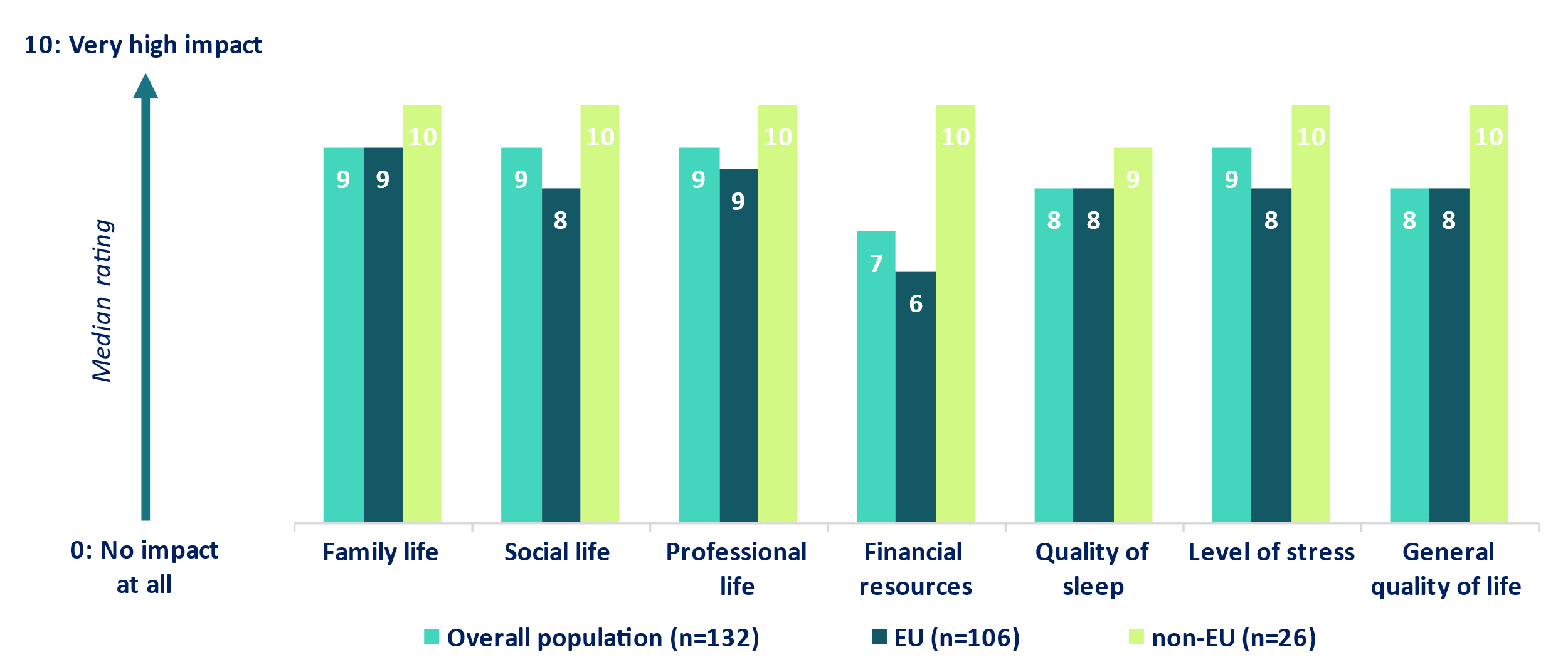


**Figure S9: Proportion of caregivers with out-of-pocket costs and ability to cover them by geographic locations**
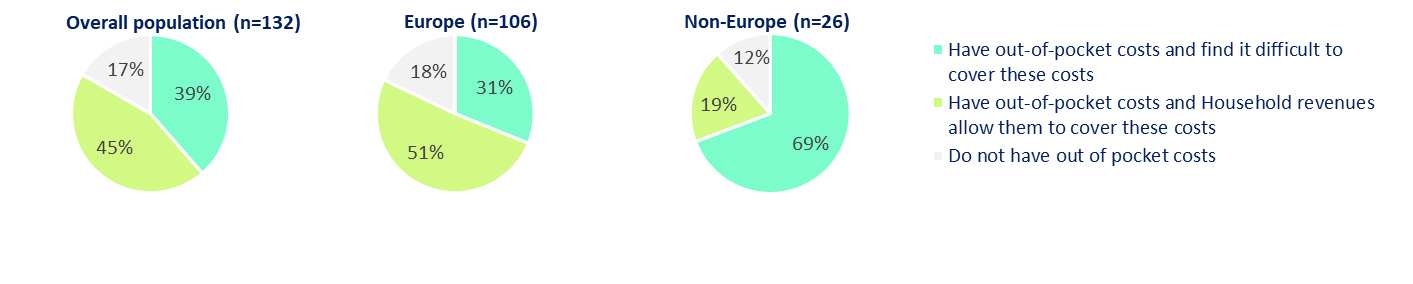


This is a Multimedia Appendix to a full manuscript published in the J Med Internet Res. For full copyright and citation information see http://dx.doi.org/10.2196/jmir.xxxx
