# Supplementary material for: Caregivers’ Perceptions of Clinical Symptoms, Disease Management, and Quality of Life Impact in Cases of Cyclin-Dependent Kinase-Like 5 Deficiency Disorder: Cross-Sectional Online Survey
Source: JMIR Form Res. 2025 Jun 10;9:e72489. doi: 10.2196/72489 (PMC12188142; doi:10.2196/72489)
Supplement: Multimedia Appendix 4 [file formative_v9i1e72489_app4.pdf]

Checklist for Reporting Results of Internet E-Surveys (CHERRIES)

Item Category	Checklist Item	Explanation
**Design**	Describe survey design	an online patient platform, Carenity. The study involved consenting adult (aged 18 years old and over) caregivers of patients who have a self-reported CDKL5 diagnosis. These caregivers participated in an online survey on CDKL5 burden without receiving any incentive for their participation in the study. Recruitment was performed through Carenity and PAGs.
**IRB (Institutional Review Board) approval and informed consent process**	IRB approval	As the primary goal of the study was to better understand the impact of the disease on patients and caregivers' lives, our study was considered to be a "satisfaction survey." This type of study typically does not require approval from an ethics committee and none was waved.
	Informed consent	Prior to participation in the study, each participant have given his/her online informed consent (opt-in). Before starting the survey, caregivers went through an introduction and information page. They were informed that the treatment of their health data is conducted based on their explicit consent, formalised by the click on the “Start” button at the bottom of the survey’s information page. They were also informed of the length of time of the survey, which data were stored and where and for how long, who the investigator was, and the purpose of the study.
	Data protection	Else Care’s platforms (carenity.com, carenity.co.uk, carenity.us, carenity.es, carenity.it, carenity.de) are hosted by AWS. Personal data were stored on AWS’s servers located in Germany (Frankfurt). An individual account (username and password) is assigned to each authorized person, who can access the data only on a private network protected by a firewall with a login and password. User permissions are defined and assigned through access control lists (ACL).
**Development and pre-testing**	Development and testing	The survey was implemented on Carenity's survey tool (proprietary tool) by a data scientists and tested by 2 other data scientists from Carenity.
**Recruitment process and description of the sample having access to the questionnaire**	Open survey versus closed survey	The survey was an open survey (open to visitors without Carenity account)
	Contact mode	Caregivers members of Carenity or CDKL5 PAGs were contacted through emails including a link redirecting to the online survey on Carenity. Reminders were also sent. Advertisements on Google or Facebook were also implemented (also redirecting to the online survey on Carenity).
	Advertising the survey	Caregivers members of Carenity or CDKL5 PAGs were contacted through emails including a link redirecting to the online survey on Carenity. Reminders were also sent. Advertisements on Google or Facebook were also implemented (also redirecting to the online survey on Carenity).

**Survey administration**	Web/E-mail	The survey was implemented on Carenity website and participants' answers were automatically registered on Carenity's database.
	Context	The Carenity platform, created in 2011, is an online patient community where both patients affected by a chronic and/or severe disease and caregivers can share their experiences, find health-related information and contribute to medical research by participating in online studies. Only patients will participate in this study.<br><br>Carenity brings together approximately 500,000 members from the EU-5 and the US, of which around 80% are patients and 20% are caregivers. Patient recruitment on Carenity is mainly done digitally, using both free and paid methods:<br><br>• Organic recruitment: Carenity website is indexed on search engines (SEO),<br><br>• Partnerships with other health websites (blogs, forums, Facebook groups, etc.),<br><br>• Paid recruitment (online campaigns): targeted online advertisements about Carenity are displayed on Google and Facebook. These paid campaigns target specific populations either through keywords or user interests. They highlight Carenity’s features (forum, health magazine, online surveys) which allow for recruitment of patients with different motivations and interests to the platform.<br><br>Partnerships and cross-promotion (e.g. dedicated newsletters, discussion topics) can also be implemented with patient organisations.<br><br>Registration on Carenity is free and the platform is accessible to all patients/caregivers over 18 years of age who have an Internet connection and an email address. Registration is accessible on computers, tablets and mobile devices and compatible with all operating systems. To register on Carenity, members must to provide an email address, a username, and their year of birth, as well as consent to the Terms of Use, formalised by a tick box, and give their explicit consent to the processing of their personal health data, after having read the terms of use via a separate tick box.<br><br>When registering on Carenity, patients can give their consent, via an opt-in, to being solicited for participation in online studies related to their condition. Upon registration, new members self-report a condition and their relationship to it (patient, caregiver or interested party) and join the corresponding community.
	Mandatory/voluntary	This survey was voluntary.

	Incentives	No incentive was offered to participants.
	Time/Date	The data collection occurred from May 25th, 2023 to December 31st, 2023.
	Randomization of items or questionnaires	Some items of the questionnaire were randomized (symptoms, type of seizure, out of pocket costs, etc.)
	Adaptive questioning	Adaptive questioning was used.
	Number of Items	The questionnaire was 40-questions long. Except specification on the questionnaire, only one question was displayed per page.
	Number of screens (pages)	Unknown
	Completeness check	Completness of the questionnaire was followed. Only completed questionnaires were included in the analyses.Consistency was checked in a quality check.
	Review step	Participants were not allowed to review and change their answers. However, participants members of Carenity had the opportunity to stop and get back where they stopped later.
**Response rates**	Unique site visitor	Not applicable
	View rate (Ratio of unique survey visitors/unique site visitors)	Unknown
	Participation rate (Ratio of unique visitors who agreed to participate/unique first survey page visitors)	Unknown
	Completion rate (Ratio of users who finished the survey/users who agreed to participate)	Unknown
**Preventing multiple entries from the same individual**	Cookies used	Cookies were used on the Carenity platform. They are kept 3 hours or 13 months depending on the participant willingness.
	IP check	No IP check was performed.
	Log file analysis	Duplicate entries were removed during quality check through check of caregivers profiles.
	Registration	Members of Carenity needed to login first to answer the survey and avoid duplicates. Members of PAGs had the opportunity answering the survey without preivous login. Possible duplicates were checked during quality check.

**Analysis**	Handling of incomplete questionnaires	Only completed questionnaires were analyzed.
	Questionnaires submitted with an atypical timestamp	Participants who answered in less than 2,6 minutes were removed from the sample during the quality check.
	Statistical correction	No statistical correction was implemented.
